# Laboratory-derived inflammatory–nutritional exploratory phenogroups and their associations with functional limitation and 30-day same-center all-cause unplanned readmission in patients with heart failure

**DOI:** 10.3389/fnut.2026.1985953

**Published:** 2026-10-09

**Authors:** Yilian Zhang, Xu Zhang, Hongbin Song, Qiyu Hu, Jingzhu Nan

**Affiliations:** 1 Department of Clinical Laboratory, Beijing Anzhen Hospital, Capital Medical University, Beijing, China; 2 Department of Clinical Laboratory, The First Medical Center of Chinese PLA, Beijing, China; 3 Department of Clinical Laboratory, The Third Medical Center of Chinese PLA, Beijing, China

**Keywords:** 30-day same-center all-cause unplanned readmission, heart failure, inflammation, nutritional assessment, nutritional status, phenotyping, functional limitation

## Abstract

**Background:**

Heart failure (HF) is characterized by marked clinical heterogeneity, while conventional left ventricular ejection fraction (LVEF)-based classification may not fully capture systemic inflammatory, nutritional, hematologic, and cardiorenal abnormalities. We aimed to identify laboratory-derived inflammatory–nutritional exploratory phenogroups in hospitalized patients with HF and evaluate their associations with functional limitation and 30-day same-center all-cause unplanned readmission.

**Methods:**

This single-center retrospective cohort included 1,588 hospitalized patients with HF, of whom 1,455 with complete data for 15 prespecified clustering variables were included in the primary analysis. K-means, Gaussian mixture models, and DBSCAN were compared, and K-means with K = 3 was selected based on internal separation, stability, cluster size, and clinical interpretability, with K = 2 used as a sensitivity analysis. Severe functional limitation was defined as NYHA class III–IV, and 30-day same-center all-cause unplanned readmission was evaluated as the short-term clinical outcome. Multivariable logistic regression with HC3 robust standard errors was used to assess these associations, with multiple-imputation and median-imputation sensitivity analyses conducted in the full cohort.

**Results:**

Three phenogroups were identified: an inflammatory–cardiorenal–nutritional vulnerability phenotype, an anemia–immune dysregulation phenotype, and a relatively preserved laboratory phenotype. LVEF was similar across groups, whereas severe functional limitation and 30-day same-center all-cause unplanned readmission differed significantly. After enhanced adjustment, compared with Cluster 3, the odds ratios for severe functional limitation were 3.814 and 5.594 for Clusters 1 and 2, respectively, and the corresponding odds ratios for 30-day same-center all-cause unplanned readmission were 1.966 and 2.616. Higher quartiles of both ALI and PNI were associated with lower odds of severe functional limitation; higher ALI quartiles and the highest PNI quartile were associated with lower odds of 30-day same-center all-cause unplanned readmission.

**Conclusion:**

Routine admission laboratory measurements identified three exploratory HF phenogroups with distinct inflammatory, nutritional, hematologic, and cardiorenal profiles. After adjustment for continuous LVEF and NT-proBNP, the two vulnerable phenogroups remained associated with severe functional limitation and 30-day same-center all-cause unplanned readmission, with directionally consistent findings in the full-cohort and K = 2 sensitivity analyses. ALI and PNI reflect laboratory-derived inflammatory–nutritional vulnerability but cannot replace formal nutritional assessment or establish incremental predictive value. These findings remain exploratory and require validation in prospective multicenter cohorts.

## 1 Introduction

Heart failure is a common clinical syndrome representing the advanced stage of multiple cardiovascular diseases and an increasing public health burden in aging populations. Global burden-of-disease studies indicate that the number of individuals living with HF continues to rise, with substantial geographic and socioeconomic disparities (1). Contemporary guideline-directed pharmacologic therapy, device therapy, cardiac rehabilitation, and multidisciplinary management have improved clinical outcomes; nevertheless, hospitalized patients with HF remain at considerable risk of functional decline, recurrent health-care utilization, and substantial resource consumption (2, 3). Early postdischarge risk is determined not only by cardiac pump function but also by comorbidities, nutritional status, and the quality of transitional care. Therefore, identifying clinically meaningful subgroups with distinct biological characteristics and short-term risks using routinely available clinical data may facilitate more individualized inpatient management and allocation of health-care resources.

Heart failure is not a single disease entity. Its clinical manifestations arise from the interplay among underlying etiology, cardiac structural and functional abnormalities, cardiorenal interactions, hematologic alterations, and inflammatory–nutritional status. Left ventricular ejection fraction remains a major component of contemporary HF classification and therapeutic decision-making; however, LVEF is a continuous and dynamic measure, and a single cutoff cannot adequately characterize differences in cardiorenal function, anemia, inflammatory burden, and systemic vulnerability (4). Even among patients within the same LVEF category, symptoms, functional status, and clinical outcomes may vary substantially. Accordingly, conventional LVEF-based stratification may fail to capture multidimensional patterns jointly reflected by routinely measured laboratory variables.

Nutritional status represents an important but frequently underrecognized component of HF heterogeneity. Appetite and dietary intake, gastrointestinal absorption, venous congestion, chronic inflammation, comorbidities, and therapeutic interventions may all influence nutritional status, whereas nutritional impairment may reduce treatment tolerance and functional recovery (5). Recent studies have demonstrated that malnutrition is common among hospitalized patients with HF and is associated with mortality and prolonged hospitalization; however, considerable heterogeneity exists across studies in both assessment methods and patient populations (6). Multidimensional nutritional assessment may provide more clinically relevant information than body weight or isolated laboratory measurements alone (7). Comprehensive assessment of patients with HF therefore requires consideration of dietary intake, laboratory biomarkers, inflammatory status, and overall clinical condition (8, 9).

Routine laboratory measurements provide an inexpensive, reproducible, and widely accessible approach to nutritional risk characterization. PNI integrates serum albumin and lymphocyte count, whereas ALI incorporates BMI, albumin, and the neutrophil-to-lymphocyte ratio to characterize inflammatory–nutritional status. Lower PNI and ALI values have previously been associated with adverse outcomes in HF (10, 11). However, individual composite indices cannot fully capture the interplay among inflammation, anemia, renal dysfunction, metabolic abnormalities, and nutritional status. Integrating routinely available laboratory measurements through unsupervised phenotyping may help identify clinically distinct subgroups with different nutritional and functional risks and provide a pragmatic basis for nutritional screening and rehabilitation-related risk assessment.

The integration of data science with clinical medicine provides new opportunities for more refined phenotyping of HF. Previous studies have used electronic health records and unsupervised machine-learning approaches to identify subgroups characterized by distinct comorbidity profiles, biological features, and clinical outcomes (12, 13). Nevertheless, existing studies are often limited by restricted LVEF ranges or limited variable availability, and laboratory-based phenotyping across the full LVEF spectrum with explicit emphasis on nutritional and inflammatory domains remains incompletely characterized. Thirty-day same-center all-cause unplanned readmission reflects early postdischarge vulnerability and is also an important measure of health-care utilization in HF (14, 15). We therefore performed unsupervised clustering based on routine admission laboratory measurements to identify inflammatory–nutritional phenotypes in hospitalized patients with HF and examined their associations with LVEF, severe functional limitation, and 30-day same-center all-cause unplanned readmission, while further evaluating the relationships of ALI and PNI with clinical outcomes.

## 2 Methods

### 2.1 Study design and study population

This was a single-center retrospective cohort study of patients hospitalized with HF at Beijing Anzhen Hospital, Capital Medical University, between June 2024 and December 2025, whose principal discharge diagnosis was HF as assessed by clinicians. For patients with multiple eligible records, only one qualifying hospitalization was retained.

The study collected biochemical panel testing; some patients underwent only individual biochemical tests and therefore had grouped missingness across multiple clustering variables. The primary clustering analysis included only patients with complete data for all 15 variables. Among 1,588 eligible patients, 1,455 had complete data for all 15 candidate clustering variables and were included in the primary complete-case clustering analysis. The remaining 133 patients were retained for full-cohort descriptions and missing-data sensitivity analyses. The detailed inclusion and exclusion process is shown in Figure 1.

**Figure 1:**
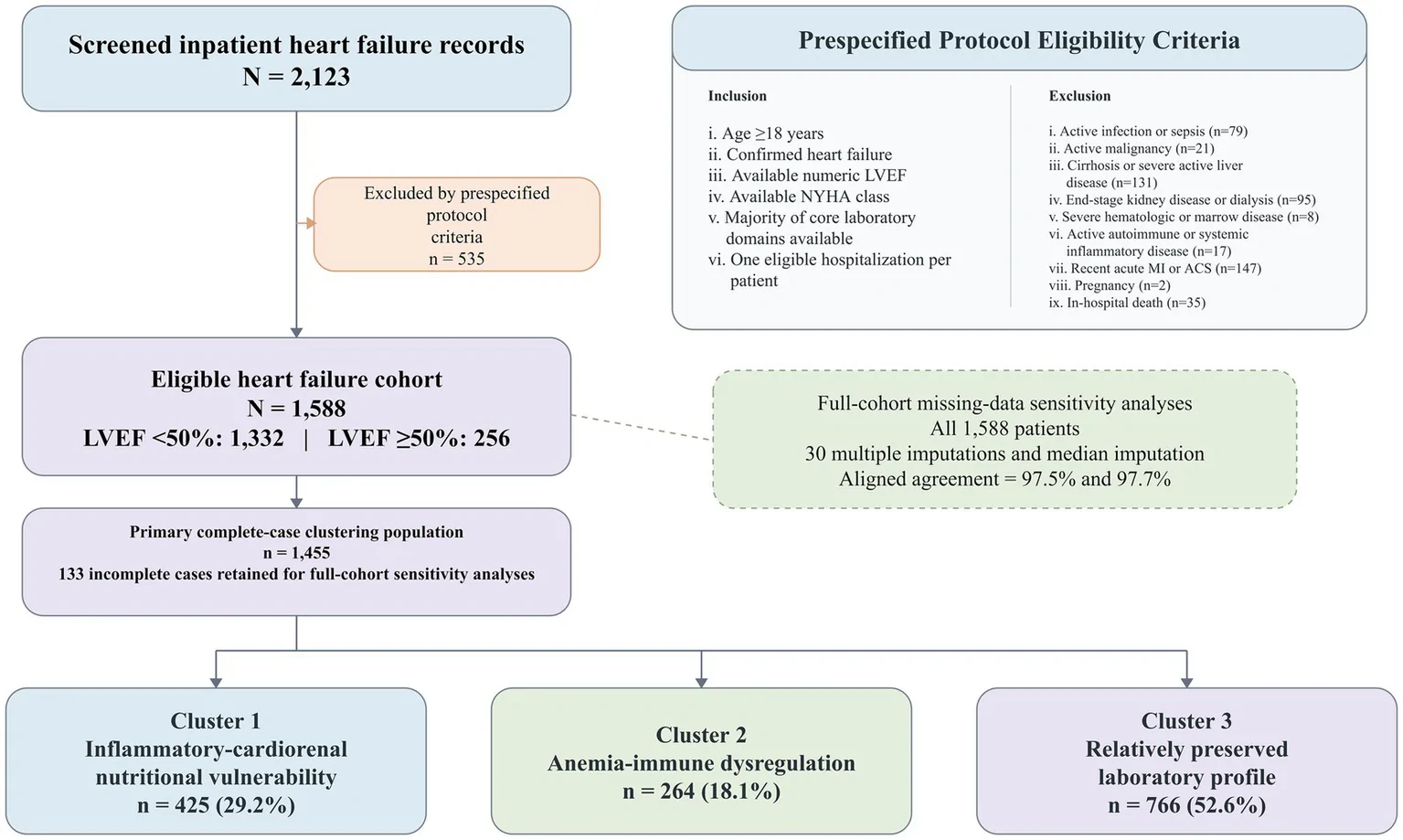
Study population and analytic cohorts. ACS, acute coronary syndrome; ARI, adjusted Rand index; LVEF, left ventricular ejection fraction; MI, myocardial infarction; NMI, normalized mutual information; NYHA, New York Heart Association.

The study was approved by the Ethics Committee of Beijing Anzhen Hospital, Capital Medical University (approval No. 2026099x). Given the retrospective use of deidentified clinical data, the requirement for informed consent was waived by the ethics committee.

### 2.2 Data sources and variable definitions

Demographic characteristics, comorbidities, cardiac functional measures, and laboratory data were obtained from the hospital information system and laboratory information system. Basic clinical variables included age, sex, BMI, hypertension, diabetes mellitus, and dyslipidemia. Valid sample sizes and missingness for all 78 source variables are provided in Supplementary Table S1.

The indicators used in this study were obtained within 48 h after admission and are usually completed before the treatment of heart failure such as major venous diuresis. The first available values of each indicator were taken within this time window. Detailed formulas and units for the derived indices are provided in Supplementary Table S2.

### 2.3 Clinical anchors and clinical outcome

NYHA class was obtained from the initial admission assessment and determined by the attending physician before clinical stabilization or adequate diuresis. Severe functional limitation was defined as NYHA functional class III or IV, whereas NYHA class I or II served as the reference category.

Thirty-day same-center all-cause unplanned readmission was defined as the first unplanned rehospitalization for any cause occurring within 30 days after discharge from the index hospitalization. Planned admissions, including prescheduled elective surgery or interventional procedures, device implantation, scheduled diagnostic evaluations or treatments, and other prearranged hospitalizations, were not counted as outcome events. Planned and unplanned readmissions were manually adjudicated by the study investigators through medical-record review. Readmission outcomes were identified only from the Beijing Anzhen Hospital information system; readmissions to other hospitals were unavailable. In-hospital deaths were excluded. All included patients had a follow-up record at this hospital more than 30 days after discharge, and no deaths within 30 days after discharge were identified. For patients with multiple qualifying unplanned readmissions within 30 days, only the first event was counted. Patients without a qualifying readmission within 30 days were classified as having no event.

Continuous LVEF, LVEF <50%, and severe functional limitation were used as post-clustering clinical anchors, whereas 30-day same-center all-cause unplanned readmission was evaluated as the short-term clinical outcome. None of these variables were included in the primary clustering model.

### 2.4 Candidate clustering variables and data preprocessing

Fifteen admission variables were prespecified for the primary clustering analysis: BMI, albumin, hemoglobin, red blood cell distribution width–coefficient of variation (RDW-CV), lymphocyte count, neutrophil count, monocyte count, high-sensitivity C-reactive protein (hs-CRP), platelet count, total cholesterol (TC), triglycerides (TG), high-density lipoprotein cholesterol (HDL-C), glucose, estimated glomerular filtration rate (eGFR), and uric acid.

Variables were selected to provide clinically meaningful coverage of anthropometric, nutritional, hematologic, inflammatory, lipid metabolic, glucose metabolic, and renal domains while minimizing substantial redundancy. The Spearman correlation structure among candidate variables is shown in Supplementary Figure S1.

Nonnegative variables with a raw skewness >1 were transformed using log(1 + x); all other variables were retained on their original scales. All 15 variables were subsequently standardized to z scores with a mean of 0 and a standard deviation of 1. The primary clustering analysis was performed in the 1,455 patients with complete data for all candidate variables.

### 2.5 Unsupervised clustering, model selection, and phenogroup labeling

K-means clustering, Gaussian mixture modeling, and DBSCAN were compared using the same standardized 15-variable matrix.

For K-means clustering, solutions with K = 2 through K = 6 were evaluated using Lloyd’s algorithm, Euclidean distance, 100 random initializations, a maximum of 1,000 iterations, and a fixed random seed of 20,260,830. For each solution, the silhouette coefficient, Calinski-Harabasz index, Davies-Bouldin index, and cluster sizes were recorded.

For GMMs, solutions with K = 2 through K = 6 were evaluated under full, tied, diagonal, and spherical covariance structures with 10 initializations. Bayesian information criterion (BIC) and internal clustering metrics were recorded. DBSCAN was screened across 40 prespecified combinations of minimum-sample requirements and empirical k-distance quantiles. Complete parameter-screening results are presented in Supplementary Table S3.

The final clustering solution was selected by jointly considering internal separation, resampling stability, cluster size, and clinical interpretability rather than relying on a single internal metric; outcome variables were not used for solution selection, and K = 3 was treated only as the primary exploratory solution. Principal component analysis (PCA) was used only for two-dimensional visualization. Phenogroups were descriptively labeled according to the relative standardized levels of the 15 clustering variables within each group.

### 2.6 Cluster stability and sensitivity analyses

Cluster stability was evaluated using 500 bootstrap resamples. For each resample, candidate clustering models were refitted at the patient level, and cluster labels were aligned with the reference solution using one-to-one matching that maximized Jaccard overlap. Mean cluster-specific and overall matched Jaccard coefficients with 95% confidence intervals (CIs) were reported. K-means solutions with K = 2 through K = 5 and tied-covariance GMM solutions with K = 3 through K = 5 were evaluated.

Missing-data sensitivity analyses included all 1,588 patients and used 30 multiple imputations and median imputation. NYHA class and the readmission outcome were excluded from the imputation process. K-means clustering with K = 3 was refitted and labels were aligned in each imputed data set, and effect estimates from multiple imputation were pooled using Rubin’s rules. A K = 2 sensitivity analysis using the same preprocessing procedures and parameters was also performed in the 1,455 complete cases (Supplementary Tables S5, S6).

### 2.7 Statistical analysis

All statistical analyses were performed using Python 3.12.3, primarily with pandas 2.3.3, NumPy 1.26.4, SciPy 1.16.3, scikit-learn 1.8.0, and statsmodels 0.14.6. Continuous variables are presented as mean ± standard deviation or median (25th–75th percentile), as appropriate, and categorical variables as counts and percentages. Comparisons between LVEF categories were performed using Welch’s *t* test or the Mann–Whitney U test for continuous variables and the chi-square or Fisher exact test for categorical variables. Comparisons across the three phenogroups were performed using the Kruskal-Wallis test for continuous variables and the chi-square test for categorical variables.

Differences in inflammatory–nutritional indices across phenogroups were descriptively evaluated using pairwise areas under the receiver operating characteristic curve (AUCs). Because several composite indices shared constituent variables with the clustering inputs, these AUCs were used only to characterize phenotypic differences and were not interpreted as independent validation or predictive performance.

Using Cluster 3 as the reference group, models for continuous LVEF and LVEF <50% were adjusted for age, sex, hypertension, diabetes mellitus, and dyslipidemia. Associations of phenogroup membership with severe functional limitation and 30-day same-center all-cause unplanned readmission were evaluated using logistic regression with HC3 robust standard errors. The severe functional limitation model was adjusted for age, sex, hypertension, diabetes mellitus, dyslipidemia, continuous LVEF, and log-transformed NT-proBNP; the readmission model was additionally adjusted for NYHA class III–IV.

ALI and PNI quartile and restricted cubic spline models used the same adjustment variables. Age-, sex-, and LVEF-stratified analyses were exploratory and were not adjusted for multiple testing. Effect estimates are reported with 95% CIs. All tests were two-sided, and *p* < 0.05 was considered the reference threshold for statistical significance.

## 3 Results

### 3.1 Study population and baseline characteristics

A total of 1,588 adult patients hospitalized with HF were included. Of these, 1,332 (83.9%) had LVEF <50% and 256 (16.1%) had LVEF ≥50%. The mean age was 60.03 ± 14.05 years, and 1,180 patients (74.3%) were men.

Severe functional limitation was present in 379 patients (23.9%), and 214 patients (13.5%) experienced 30-day same-center all-cause unplanned readmission. Severe functional limitation was more common among patients with LVEF ≥50% than among those with LVEF <50% (34.0% vs. 21.9%, *p* < 0.001), whereas 30-day same-center all-cause unplanned readmission rates were similar between the two LVEF categories (12.9% vs. 13.6%, *p* = 0.765). Additional clinical, laboratory, and inflammatory–nutritional characteristics are presented in Table 1.

**Table 1:** Baseline characteristics according to LVEF category.

Characteristic	Summary	Overall (*N* = 1,588)	LVEF <50%	LVEF > = 50%	Valid *n* (<50%)	Valid *n* (> = 50%)	*p* value
Age, years	Mean ± SD	60.03 ± 14.05	59.99 ± 14.38	60.24 ± 12.25	1,332	256	0.771
Male sex	*n* (%)	1,180 (74.3%)	983 (73.8%)	197 (77.0%)	1,332	256	0.327
BMI, kg/m^2^	Mean ± SD	23.60 ± 3.15	23.52 ± 3.14	24.00 ± 3.14	1,331	256	0.025
Hypertension	*n* (%)	886 (55.8%)	732 (55.0%)	154 (60.2%)	1,332	256	0.143
Diabetes mellitus	*n* (%)	612 (38.5%)	507 (38.1%)	105 (41.0%)	1,332	256	0.413
Dyslipidemia	*n* (%)	693 (43.6%)	573 (43.0%)	120 (46.9%)	1,332	256	0.284
LVEF, %	Mean ± SD	35.73 ± 13.72	31.00 ± 8.84	60.32 ± 6.35	1,332	256	<0.001
Severe functional limitation	*n* (%)	379 (23.9%)	292 (21.9%)	87 (34.0%)	1,332	256	<0.001
30-day same-center all-cause unplanned readmission	*n* (%)	214 (13.5%)	181 (13.6%)	33 (12.9%)	1,332	256	0.765
Hemoglobin, g/L	Mean ± SD	135.82 ± 26.22	137.01 ± 26.30	129.49 ± 24.88	1,332	251	<0.001
RDW-CV, %	Median [IQR]	13.50 [12.90, 14.80]	13.60 [12.90, 14.80]	13.40 [12.90, 14.80]	1,332	251	0.497
Lymphocyte, 10^9/L	Median [IQR]	1.46 [1.03, 1.92]	1.48 [1.05, 1.93]	1.36 [0.89, 1.85]	1,332	251	0.013
Neutrophil, 10^9/L	Median [IQR]	4.56 [3.48, 6.08]	4.52 [3.48, 5.99]	4.68 [3.52, 6.68]	1,332	251	0.042
Monocyte, 10^9/L	Median [IQR]	0.44 [0.34, 0.57]	0.43 [0.34, 0.56]	0.47 [0.35, 0.61]	1,332	251	0.027
Platelet, 10^9/L	Median [IQR]	188.00 [149.00, 233.00]	188.00 [150.00, 233.00]	190.00 [147.00, 232.00]	1,332	251	0.989
Albumin, g/L	Mean ± SD	43.06 ± 4.27	43.16 ± 4.25	42.00 ± 4.43	1,330	129	0.005
hs-CRP, mg/L	Median [IQR]	1.50 [0.68, 4.38]	1.48 [0.65, 4.25]	2.20 [0.83, 7.97]	1,331	129	0.007
Total cholesterol, mmol/L	Median [IQR]	3.70 [3.08, 4.55]	3.69 [3.08, 4.55]	3.80 [3.00, 4.79]	1,330	128	0.471
Triglycerides, mmol/L	Median [IQR]	1.33 [0.99, 1.87]	1.34 [0.99, 1.87]	1.29 [0.99, 1.92]	1,330	128	0.959
HDL-C, mmol/L	Mean ± SD	1.11 ± 0.29	1.12 ± 0.29	1.05 ± 0.30	1,330	128	0.027
Glucose, mmol/L	Median [IQR]	5.61 [4.94, 6.81]	5.63 [4.97, 6.83]	5.33 [4.74, 6.33]	1,331	129	0.02
eGFR, mL/min/1.73 m^2^	Mean ± SD	77.42 ± 24.06	77.61 ± 23.95	75.46 ± 25.20	1,330	128	0.355
Uric acid, μmol/L	Median [IQR]	337.30 [270.10, 423.75]	336.20 [269.25, 420.15]	357.15 [271.65, 493.42]	1,331	128	0.032
NT-proBNP, pg./mL	Median [IQR]	1165.00 [423.75, 3181.79]	1177.00 [438.75, 3138.99]	1140.00 [359.64, 3587.03]	1,332	256	0.755
PNI	Median [IQR]	51.30 [47.55, 54.30]	51.40 [47.60, 54.30]	49.85 [46.50, 53.30]	1,330	129	0.042
CONUT score	Mean ± SD	2.46 ± 1.56	2.45 ± 1.54	2.56 ± 1.75	1,329	128	0.489
HALP	Median [IQR]	47.06 [29.92, 66.78]	47.81 [30.98, 66.80]	39.58 [18.92, 66.16]	1,330	129	0.011
hsCAR	Median [IQR]	0.03 [0.02, 0.11]	0.03 [0.01, 0.10]	0.05 [0.02, 0.19]	1,330	129	0.005
ALI	Median [IQR]	33.41 [19.97, 50.95]	33.41 [20.19, 50.33]	33.43 [15.10, 57.17]	1,329	129	0.836

ALI, advanced lung cancer inflammation index; BMI, body mass index; CALLY, C-reactive protein-albumin-lymphocyte score; CONUT, Controlling Nutritional Status; eGFR, estimated glomerular filtration rate; HALP, hemoglobin-albumin-lymphocyte-platelet; HDL-C, high-density lipoprotein cholesterol; hsCAR, high-sensitivity C-reactive protein-to-albumin ratio; hs-CRP, high-sensitivity C-reactive protein; IQR, interquartile range; LVEF, left ventricular ejection fraction; NPS, Naples prognostic score; NT-proBNP, N-terminal pro-B-type natriuretic peptide; PNI, prognostic nutritional index; RAR, red cell distribution width-to-albumin ratio; RDW-CV, red blood cell distribution width-coefficient of variation; SD, standard deviation.

### 3.2 Candidate variables, preprocessing, and selection of the clustering solution

Fifteen admission variables were prespecified for clustering, covering anthropometric, nutritional, hematologic, inflammatory, lipid metabolic, glucose metabolic, and renal domains. Missingness ranged from 0.1 to 8.2%. Nonnegative variables with raw skewness >1 were transformed using log(1 + x), after which all variables were standardized to z scores. A total of 1,455 patients had complete data for all 15 variables and were included in the primary complete-case analysis (Table 2). The Spearman correlation matrix of the candidate variables is shown in Supplementary Figure S1. The proportions of complete cases were 99.6% among patients with LVEF <50 and 50.0% among those with LVEF ≥50%, indicating differential complete-case selection (Supplementary Table S7).

**Table 2:** Candidate clustering variables, data preprocessing, and inclusion status.

Variable	Valid *n*	Missing (%)	Raw skewness	Transformation
BMI	1,587	0.1	0.431	None
Albumin	1,459	8.1	−0.757	None
Hemoglobin	1,583	0.3	−0.397	None
RDW-CV	1,583	0.3	2.525	log1p
Lymphocyte	1,583	0.3	18.511	log1p
Neutrophil	1,583	0.3	2.737	log1p
Monocyte	1,583	0.3	2.845	log1p
hs-CRP	1,460	8.1	7.608	log1p
Platelet	1,583	0.3	1.552	log1p
TC	1,458	8.2	1.162	log1p
TG	1,458	8.2	10.516	log1p
HDL-C	1,458	8.2	0.913	None
Glucose	1,460	8.1	2.479	log1p
eGFR	1,458	8.2	−0.417	None
Uric acid	1,459	8.1	1.244	log1p

BMI, body mass index; eGFR, estimated glomerular filtration rate; HDL-C, high-density lipoprotein cholesterol; hs-CRP, high-sensitivity C-reactive protein; LVEF, left ventricular ejection fraction; NT-proBNP, N-terminal pro-B-type natriuretic peptide; RDW-CV, red blood cell distribution width-coefficient of variation; TC, total cholesterol; TG, triglycerides.

The clustering-method comparison is shown in Figure 2, with complete parameter-screening results provided in Supplementary Table S3. For K-means with K = 3, the silhouette coefficient was 0.107, the mean bootstrap matched Jaccard coefficient was 0.823, and the mean Jaccard coefficient of the least stable cluster was 0.784 (Supplementary Table S4). The lowest BIC for GMM occurred at K = 5; however, the smallest subgroup comprised only 3.64% of participants. DBSCAN failed to identify a valid multi-cluster solution across all 40 parameter settings.

**Figure 2:**
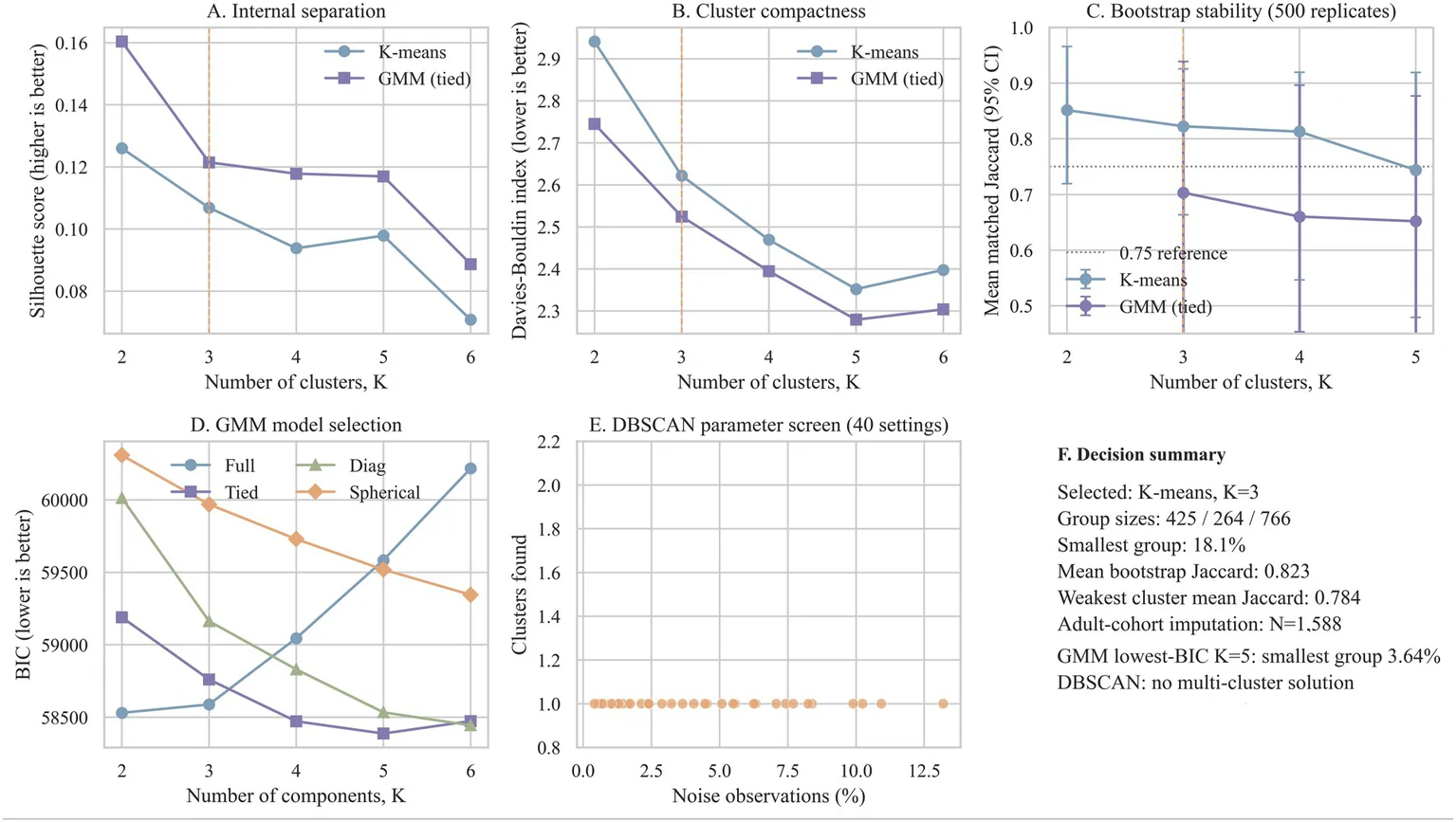
Comparison of clustering methods and selection of an exploratory K = 3 solution. **(A)** Shows the silhouette coefficient for different numbers of clusters; **(B)** Shows the Davies-Bouldin index; **(C)** Shows the matched Jaccard coefficient and 95% CI obtained from 500 bootstrap repetitions; **(D)** Shows the BIC for GMMs with different covariance structures; **(E)** Shows the results of DBSCAN parameter screening; **(F)** Summarizes the final selection. Based on a comprehensive evaluation of internal separation, cluster size, stability, and interpretability, K-means with K = 3 was selected as the primary exploratory solution. BIC, Bayesian information criterion; CI, confidence interval; DBSCAN, density-based spatial clustering of applications with noise; GMM, Gaussian mixture model; K, number of clusters.

Considering internal separation, stability, subgroup size, and clinical interpretability, K-means clustering with K = 3 was selected as the primary exploratory solution. After multiple imputation and median imputation, agreement of the K = 3 labels with the primary analysis was 97.5 and 97.7%, respectively, and the directions of association with both outcomes were consistent (Supplementary Table S5). The K = 2 solution had a silhouette coefficient of 0.126, higher than the 0.107 observed for K = 3, but the directions of association with both outcomes were consistent (Supplementary Table S6). K = 3 was therefore retained as the clinically interpretable primary exploratory solution.

### 3.3 Identification and characteristics of the three exploratory HF phenogroups

In the two-dimensional PCA projection, principal components 1 and 2 explained 14.7 and 12.0% of the total variance, respectively. The three phenogroups showed different relative distributions in the two-dimensional space, although substantial overlap remained. Therefore, the PCA plot was used for visualization rather than to define deterministic cluster boundaries.

K-means clustering with K = 3 identified three exploratory HF phenogroups. Cluster 1, comprising 425 patients (29.2%), was characterized as an inflammatory–cardiorenal–nutritional vulnerability phenotype. This group was characterized by higher hs-CRP, glucose, and uric acid levels and lower albumin, eGFR, and HDL-C levels.

Cluster 2 included 264 patients (18.1%) and was characterized as an anemia–immune dysregulation phenotype, with lower hemoglobin, lymphocyte count, and platelet count and higher RDW-CV, neutrophil count, and monocyte count (Figure 3).

**Figure 3:**
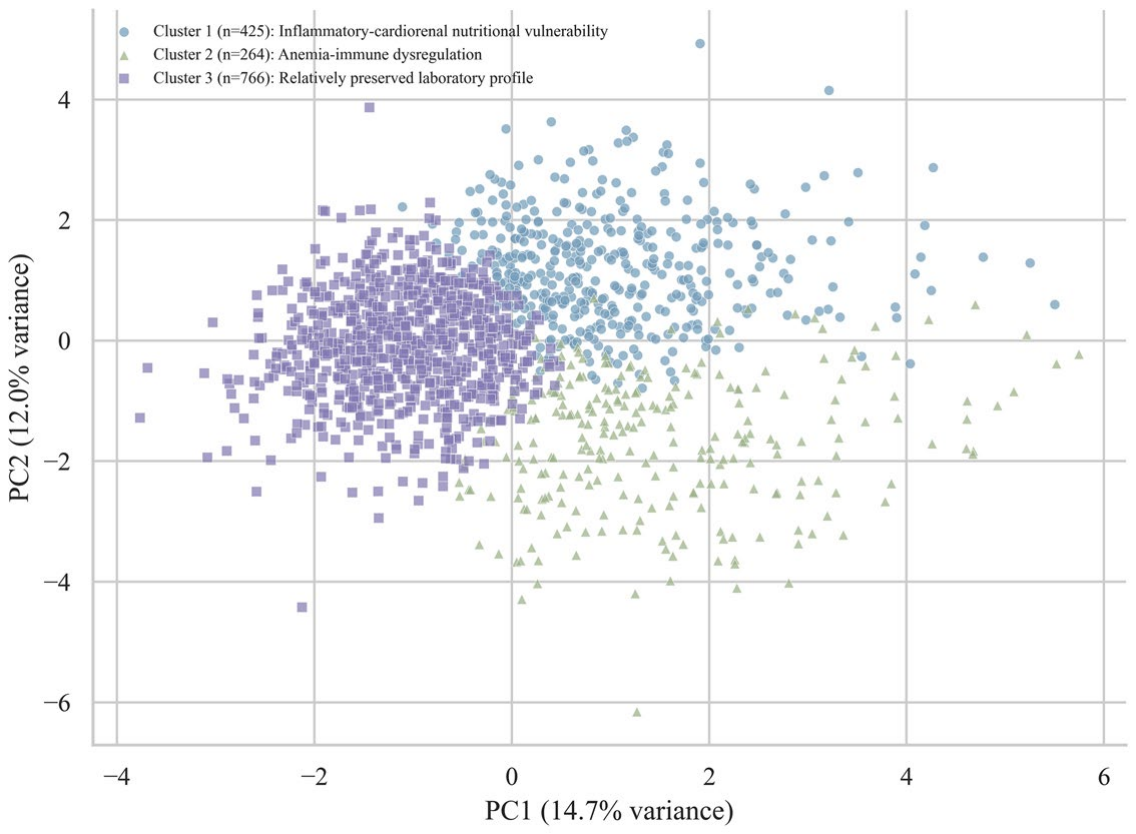
Principal component analysis visualization of the three exploratory phenogroups. The scatter plots are based on the primary complete-case cohort (*n* = 1,455). PC1 and PC2 explain 14.7 and 12.0% of the variance, respectively. PCA, principal component analysis; PC1, first principal component; PC2, second principal component.

Cluster 3 comprised 766 patients (52.6%) and represented a relatively preserved laboratory phenotype.

Sex distribution, BMI, and the prevalence of hypertension, diabetes mellitus, and dyslipidemia were similar across phenogroups. In contrast, age, severe functional limitation, 30-day same-center all-cause unplanned readmission, and most laboratory and inflammatory–nutritional indices differed significantly across groups (Table 3).

**Table 3:** Clinical, laboratory, and 30-day same-center all-cause unplanned readmission characteristics across the three exploratory phenogroups.

Characteristic	Summary	Overall (*N* = 1,455)	Cluster 1 (*n* = 425)	Cluster 2 (*n* = 264)	Cluster 3 (*n* = 766)	*P* value
Age, years	Mean ± SD	60.00 ± 14.20	61.44 ± 14.21	60.33 ± 12.62	59.09 ± 14.65	0.016
Male sex	n (%)	1,076 (74.0%)	309 (72.7%)	199 (75.4%)	568 (74.2%)	0.727
BMI, kg/m^2^	Mean ± SD	23.55 ± 3.13	23.40 ± 3.09	23.43 ± 3.16	23.68 ± 3.14	0.183
Hypertension	n (%)	807 (55.5%)	233 (54.8%)	154 (58.3%)	420 (54.8%)	0.584
Diabetes mellitus	n (%)	558 (38.4%)	167 (39.3%)	108 (40.9%)	283 (36.9%)	0.465
Dyslipidemia	n (%)	631 (43.4%)	186 (43.8%)	114 (43.2%)	331 (43.2%)	0.981
LVEF, %	Mean ± SD	33.54 ± 11.94	33.98 ± 12.63	33.73 ± 12.10	33.23 ± 11.48	0.954
Severe functional limitation	n (%)	371 (25.5%)	155 (36.5%)	119 (45.1%)	97 (12.7%)	<0.001
30-day same-center all-cause unplanned readmission	n (%)	196 (13.5%)	71 (16.7%)	55 (20.8%)	70 (9.1%)	<0.001
Hemoglobin, g/L	Mean ± SD	136.56 ± 26.33	139.71 ± 23.28	104.67 ± 21.79	145.81 ± 20.30	<0.001
RDW-CV, %	Median [IQR]	13.60 [13.00, 14.90]	13.50 [13.00, 14.80]	15.90 [14.60, 17.12]	13.30 [12.80, 13.90]	<0.001
Lymphocyte, 10^9/L	Median [IQR]	1.47 [1.05, 1.93]	1.53 [1.13, 1.89]	0.81 [0.52, 1.14]	1.69 [1.27, 2.13]	<0.001
Neutrophil, 10^9/L	Median [IQR]	4.53 [3.48, 5.99]	4.31 [3.46, 5.43]	7.47 [4.97, 10.60]	4.19 [3.29, 5.36]	<0.001
Monocyte, 10^9/L	Median [IQR]	0.43 [0.34, 0.56]	0.44 [0.35, 0.53]	0.53 [0.40, 0.71]	0.41 [0.33, 0.53]	<0.001
Platelet, 10^9/L	Median [IQR]	189.00 [150.00, 234.00]	193.00 [155.00, 239.00]	147.50 [95.75, 205.75]	195.00 [160.25, 237.00]	<0.001
Albumin, g/L	Mean ± SD	43.06 ± 4.28	40.05 ± 4.36	43.49 ± 3.93	44.58 ± 3.39	<0.001
hs-CRP, mg/L	Median [IQR]	1.50 [0.68, 4.39]	5.45 [2.38, 14.70]	1.40 [0.69, 3.64]	0.92 [0.50, 1.85]	<0.001
Total cholesterol, mmol/L	Median [IQR]	3.69 [3.08, 4.55]	3.62 [3.00, 4.37]	3.83 [3.03, 4.84]	3.69 [3.12, 4.55]	0.172
Triglycerides, mmol/L	Median [IQR]	1.33 [0.99, 1.87]	1.43 [1.07, 2.10]	1.40 [1.02, 1.96]	1.23 [0.95, 1.73]	<0.001
HDL-C, mmol/L	Mean ± SD	1.11 ± 0.29	0.96 ± 0.24	1.08 ± 0.27	1.20 ± 0.29	<0.001
Glucose, mmol/L	Median [IQR]	5.60 [4.94, 6.81]	6.02 [5.11, 8.09]	5.54 [4.85, 6.68]	5.49 [4.90, 6.33]	<0.001
eGFR, mL/min/1.73 m^2^	Mean ± SD	77.41 ± 24.04	61.60 ± 24.09	78.27 ± 24.02	85.89 ± 19.17	<0.001
Uric acid, μmol/L	Median [IQR]	337.30 [270.00, 423.75]	431.60 [351.60, 537.10]	343.80 [273.25, 428.15]	298.50 [243.80, 356.23]	<0.001
NT-proBNP, pg./mL	Median [IQR]	1192.15 [443.77, 3236.64]	1337.00 [533.00, 3448.00]	1254.00 [550.99, 4395.75]	1080.52 [387.25, 2829.25]	0.002
PNI	Median [IQR]	51.30 [47.55, 54.30]	48.55 [44.60, 52.00]	48.25 [45.08, 51.30]	53.10 [50.50, 56.14]	<0.001
CONUT score	Mean ± SD	2.46 ± 1.56	2.62 ± 1.69	3.50 ± 1.49	2.02 ± 1.30	<0.001
HALP	Median [IQR]	47.06 [29.92, 66.78]	44.52 [29.59, 61.62]	23.67 [13.59, 37.68]	55.03 [40.26, 75.96]	<0.001
hsCAR	Median [IQR]	0.03 [0.02, 0.11]	0.14 [0.06, 0.39]	0.03 [0.02, 0.09]	0.02 [0.01, 0.04]	<0.001
ALI	Median [IQR]	33.42 [19.98, 50.93]	33.36 [20.53, 47.05]	11.73 [5.65, 21.08]	41.45 [28.96, 58.43]	<0.001
CALLY	Median [IQR]	38.18 [12.29, 98.74]	11.35 [3.57, 28.37]	22.66 [6.83, 53.60]	77.93 [34.50, 152.40]	<0.001
NPS	Mean ± SD	2.16 ± 1.06	2.41 ± 1.10	2.76 ± 0.75	1.81 ± 1.00	<0.001
RAR	Median [IQR]	0.32 [0.29, 0.36]	0.34 [0.31, 0.38]	0.37 [0.33, 0.41]	0.30 [0.28, 0.32]	<0.001

Three exploratory phenogroups were identified through K-means clustering of 1,455 complete cases (K = 3, random seed 20,260,830, n_init = 100). Cluster 1 represents the inflammatory–cardiorenal–nutritional vulnerability phenotype; Cluster 2 represents the anemia–immune dysregulation phenotype; and Cluster 3 represents the relatively preserved laboratory phenotype. ALI, advanced lung cancer inflammation index; BMI, body mass index; CALLY, C-reactive protein-albumin-lymphocyte score; CONUT, Controlling Nutritional Status; eGFR, estimated glomerular filtration rate; HALP, hemoglobin-albumin-lymphocyte-platelet; HDL-C, high-density lipoprotein cholesterol; hsCAR, high-sensitivity C-reactive protein-to-albumin ratio; hs-CRP, high-sensitivity C-reactive protein; IQR, interquartile range; LVEF, left ventricular ejection fraction; NPS, Naples prognostic score; NT-proBNP, N-terminal pro-B-type natriuretic peptide; PNI, prognostic nutritional index; RAR, red cell distribution width-to-albumin ratio; RDW-CV, red blood cell distribution width-coefficient of variation; SD, standard deviation.

### 3.4 Adjusted associations of phenogroups with clinical anchors and clinical outcome

Using Cluster 3 as the reference group and adjusting for age, sex, hypertension, diabetes mellitus, and dyslipidemia, no significant differences in continuous LVEF were observed for Cluster 1 or Cluster 2.

Cluster 1 was associated with lower odds of LVEF <50% (OR, 0.574; 95% CI, 0.380–0.867; *p* = 0.008), whereas the corresponding association for Cluster 2 did not reach conventional statistical significance (OR, 0.623; 95% CI, 0.385–1.008; *p* = 0.054).

After enhanced adjustment, compared with Cluster 3, the ORs for severe functional limitation were 3.814 (95% CI, 2.813–5.170) for Cluster 1 and 5.594 (95% CI, 3.928–7.965) for Cluster 2. The corresponding ORs for 30-day same-center all-cause unplanned readmission were 1.966 (95% CI, 1.364–2.835) and 2.616 (95% CI, 1.740–3.932), respectively (Table 4).

**Table 4:** Adjusted associations of exploratory phenogroups with clinical anchors and 30-day same-center all-cause unplanned readmission.

Outcome	Comparison	Adjusted effect (95% CI)	*P* value
LVEF (%)	Cluster 1 vs. Cluster 3	0.728 (−0.732 to 2.189)	0.328
LVEF (%)	Cluster 2 vs. Cluster 3	0.413 (−1.250 to 2.077)	0.626
LVEF <50%	Cluster 1 vs. Cluster 3	0.574 (0.380 to 0.867)	0.008
LVEF <50%	Cluster 2 vs. Cluster 3	0.623 (0.385 to 1.008)	0.054
Severe functional limitation	Cluster 1 vs. Cluster 3	3.814 (2.813 to 5.170)	<0.001
Severe functional limitation	Cluster 2 vs. Cluster 3	5.594 (3.928 to 7.965)	<0.001
30-day same-center all-cause unplanned readmission	Cluster 1 vs. Cluster 3	1.966 (1.364 to 2.835)	<0.001
30-day same-center all-cause unplanned readmission	Cluster 2 vs. Cluster 3	2.616 (1.740 to 3.932)	<0.001

Cluster 3 served as the reference group. Models for LVEF-related outcomes were adjusted for age, sex, hypertension, diabetes mellitus, and dyslipidemia. The severe functional limitation model used logistic regression with HC3 robust standard errors and was adjusted for age, sex, hypertension, diabetes mellitus, dyslipidemia, continuous LVEF, and log-transformed NT-proBNP; the readmission model was additionally adjusted for NYHA class III–IV. CI, confidence interval; LVEF, left ventricular ejection fraction; OR, odds ratio.

### 3.5 Distribution and descriptive discrimination of inflammatory–nutritional indices across phenogroups

Among the 11 inflammatory–nutritional indices evaluated, ALI showed marked descriptive differences across phenogroups, with the highest mean pairwise AUC of 0.790 (95% CI, 0.773–0.808) and a minimum pairwise AUC of 0.625 (95% CI, 0.590–0.661). For the traditional PNI, the three-group effect size was 0.236 (95% CI, 0.203–0.278), and the mean pairwise AUC was 0.696 (95% CI, 0.680–0.715).

Because ALI, PNI, and several other composite indices share constituent variables with the clustering inputs, these findings were interpreted only as descriptive characterization of inflammatory–nutritional differences across phenogroups and not as independent phenotype validation or predictive performance (Table 5). Formulas and units for all inflammatory–nutritional indices are provided in Supplementary Table S2.

**Table 5:** Distribution and descriptive pairwise discrimination of inflammatory–nutritional indices across the exploratory phenogroups.

Index	Cluster medians (C1/C2/C3)	Epsilon-squared (95% CI)	Mean pairwise AUC (95% CI)
ALI	33.36/11.73/41.45	0.289 (0.256 to 0.323)	0.790 (0.773 to 0.808)
PNI	48.55/48.25/53.10	0.236 (0.203 to 0.278)	0.696 (0.680 to 0.715)
CALLY	11.35/22.66/77.93	0.324 (0.285 to 0.366)	0.746 (0.728 to 0.767)
RAR	0.34/0.37/0.30	0.275 (0.233 to 0.314)	0.740 (0.720 to 0.759)
HALP	44.52/23.67/55.03	0.199 (0.166 to 0.238)	0.737 (0.716 to 0.756)
hsCAR	0.14/0.03/0.02	0.298 (0.264 to 0.336)	0.745 (0.724 to 0.764)
LCR	0.29/0.52/1.74	0.310 (0.269 to 0.353)	0.739 (0.720 to 0.761)
NAR	0.11/0.17/0.09	0.150 (0.118 to 0.187)	0.708 (0.687 to 0.727)
NPS	2.00/3.00/2.00	0.131 (0.104 to 0.163)	0.668 (0.649 to 0.686)
CONUT score	2.00/4.00/2.00	0.127 (0.098 to 0.161)	0.683 (0.664 to 0.702)
MAR	0.01/0.01/0.01	0.060 (0.038 to 0.091)	0.621 (0.596 to 0.643)

ε^2^ is based on the Kruskal-Wallis test; both the AUC and the confidence intervals for the effect size were estimated using 500 bootstrap repetitions. ALI, advanced lung cancer inflammation index; AUC, area under the receiver operating characteristic curve; CALLY, C-reactive protein-albumin-lymphocyte score; CI, confidence interval; CONUT, Controlling Nutritional Status; HALP, hemoglobin-albumin-lymphocyte-platelet; hsCAR, high-sensitivity C-reactive protein-to-albumin ratio; LCR, lymphocyte-to-C-reactive protein ratio; MAR, monocyte-to-albumin ratio; NAR, neutrophil-to-albumin ratio; NPS, Naples prognostic score; PNI, prognostic nutritional index; RAR, red cell distribution width-to-albumin ratio.

### 3.6 Associations of ALI and PNI quartiles with severe functional limitation and 30-day same-center all-cause unplanned readmission

Using the lowest quartile (Q1) as the reference group, after enhanced adjustment, progressively higher quartiles of ALI and PNI were associated with lower odds of severe functional limitation (all *p* < 0.001).

For 30-day same-center all-cause unplanned readmission, ALI quartiles Q2–Q4 were associated with lower odds than Q1, whereas for PNI only Q4 was associated with lower odds than Q1 (OR, 0.533; 95% CI, 0.335–0.849; *p* = 0.008) (Table 6).

**Table 6:** Associations of ALI and PNI quartiles with severe functional limitation and 30-day same-center all-cause unplanned readmission.

Index	Outcome	Quartile	*N* (events)	Model 1		Model 2		Model 3	
				OR (95% CI)	*P* value	OR (95% CI)	*P* value	OR (95% CI)	*P* value
ALI	Severe functional limitation	Q1	364 (184)	Reference		Reference		Reference	
ALI	Severe functional limitation	Q2	364 (78)	0.267 (0.193 to 0.369)	<0.001	0.267 (0.193 to 0.369)	<0.001	0.278 (0.195 to 0.395)	<0.001
ALI	Severe functional limitation	Q3	363 (59)	0.190 (0.134 to 0.268)	<0.001	0.191 (0.135 to 0.270)	<0.001	0.173 (0.119 to 0.251)	<0.001
ALI	Severe functional limitation	Q4	364 (50)	0.156 (0.108 to 0.224)	<0.001	0.159 (0.110 to 0.229)	<0.001	0.148 (0.099 to 0.220)	<0.001
ALI	30-day same-center all-cause unplanned readmission	Q1	364 (72)	Reference		Reference		Reference	
ALI	30-day same-center all-cause unplanned readmission	Q2	364 (42)	0.529 (0.350 to 0.799)	0.002	0.533 (0.352 to 0.806)	0.003	0.549 (0.354 to 0.851)	0.007
ALI	30-day same-center all-cause unplanned readmission	Q3	363 (47)	0.603 (0.404 to 0.900)	0.013	0.605 (0.406 to 0.903)	0.014	0.617 (0.407 to 0.937)	0.023
ALI	30-day same-center all-cause unplanned readmission	Q4	364 (35)	0.431 (0.280 to 0.666)	<0.001	0.435 (0.280 to 0.674)	<0.001	0.451 (0.284 to 0.715)	<0.001
PNI	Severe functional limitation	Q1	369 (180)	Reference		Reference		Reference	
PNI	Severe functional limitation	Q2	363 (93)	0.362 (0.265 to 0.494)	<0.001	0.365 (0.267 to 0.498)	<0.001	0.373 (0.266 to 0.525)	<0.001
PNI	Severe functional limitation	Q3	359 (54)	0.186 (0.130 to 0.265)	<0.001	0.188 (0.132 to 0.267)	<0.001	0.179 (0.123 to 0.261)	<0.001
PNI	Severe functional limitation	Q4	364 (44)	0.144 (0.099 to 0.210)	<0.001	0.149 (0.102 to 0.218)	<0.001	0.148 (0.099 to 0.222)	<0.001
PNI	30-day same-center all-cause unplanned readmission	Q1	369 (64)	Reference		Reference		Reference	
PNI	30-day same-center all-cause unplanned readmission	Q2	363 (52)	0.797 (0.535 to 1.187)	0.264	0.800 (0.537 to 1.193)	0.275	0.828 (0.552 to 1.240)	0.359
PNI	30-day same-center all-cause unplanned readmission	Q3	359 (46)	0.700 (0.465 to 1.056)	0.089	0.705 (0.467 to 1.065)	0.097	0.739 (0.482 to 1.135)	0.167
PNI	30-day same-center all-cause unplanned readmission	Q4	364 (34)	0.491 (0.315 to 0.766)	0.002	0.506 (0.324 to 0.790)	0.003	0.533 (0.335 to 0.849)	0.008

Quartiles were defined as the 25th, 50th, and 75th percentiles in the complete-case cohort for the primary analysis (*N* = 1,455), with values equal to the cutoff points assigned to the lower quartile; the ALI cutoff points were 19.98, 33.42, and 50.93, and the PNI cutoff points were 47.55, 51.30, and 54.30, with Q1 as the reference. Model 1 was unadjusted; Model 2 was adjusted for age and sex; Model 3 was further adjusted for hypertension, diabetes mellitus, dyslipidemia, continuous LVEF, and log-transformed NT-proBNP, with NYHA class III–IV additionally included in the readmission model. Logistic regression used HC3 robust standard errors. All *p* values are two-sided. ALI, advanced lung cancer inflammation index; CI, confidence interval; OR, odds ratio; PNI, prognostic nutritional index.

### 3.7 Restricted cubic spline associations of inflammatory–nutritional indices with clinical outcomes

After enhanced adjustment, restricted cubic spline analyses demonstrated significant overall and nonlinear associations of both ALI and PNI with severe functional limitation (all overall and nonlinear *p* < 0.001) (Figure 4).

**Figure 4:**
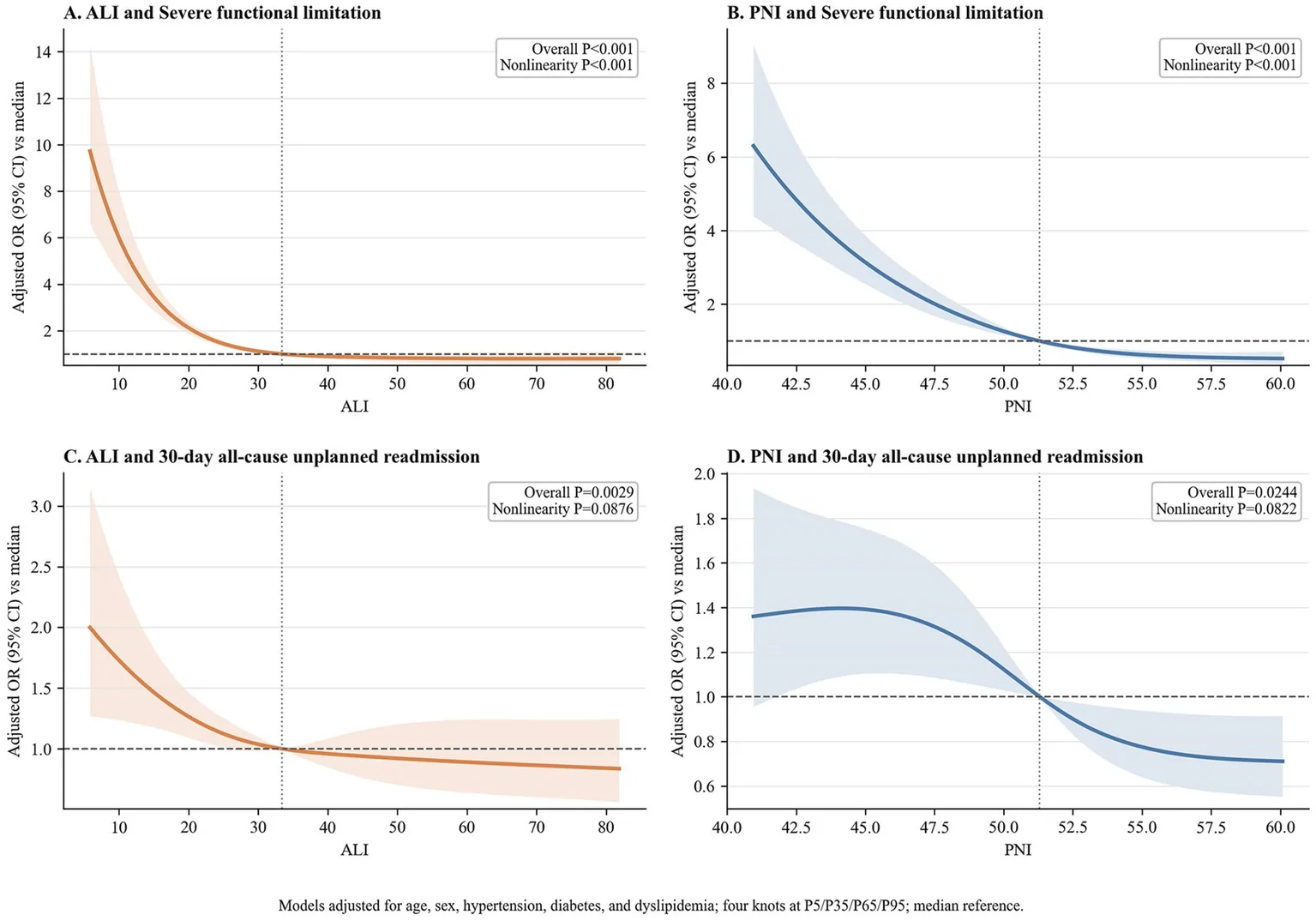
Restricted cubic spline associations of ALI and PNI with severe functional limitation and 30-day same-center all-cause unplanned readmission. Panels **(A,B)** show the associations of ALI and PNI, respectively, with severe functional limitation; panels **(C,D)** show their associations with 30-day same-center all-cause unplanned readmission. Solid lines represent adjusted odds ratios (ORs), and shaded areas indicate 95% confidence intervals (CIs); horizontal dashed lines indicate OR = 1, and vertical dashed lines indicate the median reference value for the corresponding index. Models used logistic regression with HC3 robust standard errors. The severe functional limitation models were adjusted for age, sex, hypertension, diabetes mellitus, dyslipidemia, continuous LVEF, and log-transformed NT-proBNP; the readmission models were additionally adjusted for NYHA class III–IV. Four knots were placed at P5, P35, P65, and P95. ALI, advanced lung cancer inflammation index; CI, confidence interval; OR, odds ratio; PNI, prognostic nutritional index; RCS, restricted cubic spline.

For 30-day same-center all-cause unplanned readmission, the overall and nonlinear *p* values were 0.011 and 0.133 for ALI and 0.046 and 0.115 for PNI, respectively.

Age-, sex-, and LVEF-stratified results were not adjusted for multiple testing and were considered hypothesis-generating only (Supplementary Figure S2).

## 4 Discussion

In this study, three exploratory phenogroups were identified among 1,455 hospitalized patients with HF using routine admission laboratory measurements. After enhanced adjustment, compared with the relatively preserved laboratory phenotype, the inflammatory–cardiorenal–nutritional vulnerability phenotype had ORs of 3.814 for severe functional limitation and 1.966 for 30-day same-center all-cause unplanned readmission, whereas the corresponding ORs for the anemia–immune dysregulation phenotype were 5.594 and 2.616. LVEF was similar across the three phenogroups, and after further adjustment for continuous LVEF and NT-proBNP, both vulnerable phenogroups remained associated with both outcomes, suggesting that phenogroup membership provided complementary association information beyond LVEF. After enhanced adjustment, ALI was associated with both outcomes, whereas the association of PNI with readmission was mainly observed in the highest quartile. These exploratory findings support the need for further comprehensive assessment.

The enhanced models and full-cohort sensitivity analyses yielded directionally consistent associations after adjustment for continuous LVEF and NT-proBNP. These findings indicate that routine laboratory measurements may capture complementary dimensions of systemic vulnerability beyond LVEF, but they do not establish incremental predictive performance or clinical utility.

Our findings are consistent with previous HF phenotyping studies demonstrating that data-driven subgroups based on comorbidities and laboratory variables may provide clinically relevant information beyond conventional LVEF classification (16). In HF with preserved ejection fraction, acute HF, and critically ill cardiac populations, phenogroups characterized by inflammation, cardiorenal dysfunction, anemia, and iron deficiency have repeatedly been identified (17–19). Similar multidimensional heterogeneity has been demonstrated using different clustering approaches and therapeutic cohorts (20, 21). The present study integrated these features into three exploratory phenogroups and described their associations with functional limitation and 30-day same-center all-cause unplanned readmission within a single framework. The low silhouette coefficient and overlap in the PCA projection indicate that these phenogroups should be interpreted as data-driven partitions within a continuum of clinical heterogeneity rather than as clearly separated biological entities; the K = 2 sensitivity analysis yielded directionally consistent outcome associations (Supplementary Table S6).

The anemia–immune dysregulation phenotype was characterized by lower hemoglobin, lymphocyte count, and platelet count, together with higher RDW-CV, neutrophil count, and monocyte count. Anemia is strongly associated with adverse clinical outcomes in HF (22), and RDW may reflect the combined effects of inflammation, nutritional status, renal dysfunction, and impaired erythropoiesis (23). Anemia may also reduce tissue oxygen delivery and exercise tolerance (24). Alterations in neutrophil, monocyte, and lymphocyte profiles reflect persistent inflammatory and immune activation and have been associated with adverse cardiovascular outcomes (25, 26). Collectively, these abnormalities characterize a state of anemia, inflammation, and immune dysregulation that may help explain the greater functional limitation and higher early readmission rate observed in this phenogroup.

The inflammatory–cardiorenal–nutritional vulnerability phenotype was characterized by higher hs-CRP, uric acid, and glucose levels and lower albumin, eGFR, and HDL-C levels. Venous congestion, impaired renal perfusion, and metabolic abnormalities may interact to increase systemic disease burden (27). Low albumin may reflect impaired nutritional status, inflammatory activity, and altered volume status (28), whereas elevated uric acid may indicate greater metabolic and oxidative stress (29). The combined pattern of higher hs-CRP and lower albumin further highlights the close interplay between inflammation and nutritional vulnerability (30). However, albumin and BMI are also influenced by inflammation, volume status, and other clinical factors and cannot independently diagnose malnutrition. This phenogroup therefore reflects a clinically coherent pattern of inflammatory activation, cardiorenal dysfunction, metabolic burden, and nutritional vulnerability.

Lower PNI has previously been associated with adverse outcomes in HF (11), and recent studies have also reported associations between ALI and HF outcomes (31, 32). However, ALI and PNI reflect laboratory-derived inflammatory–nutritional vulnerability. After enhanced adjustment, both indices were associated with severe functional limitation; ALI was associated with 30-day same-center all-cause unplanned readmission, whereas the association for PNI was mainly observed in the highest quartile. Because their component variables overlap with the clustering inputs and this was an observational study, these results do not provide independent validation or evidence of incremental predictive value, nor do they establish independent biological pathways or causal effects. ALI and PNI cannot replace formal nutritional assessments such as GLIM, SGA, or MNA. Their inverse associations with severe functional limitation were stronger among patients aged <65 years, possibly because functional status in older patients is also influenced by frailty, multimorbidity, and sarcopenia; however, this subgroup analysis was exploratory and should not be used to define age-specific thresholds.

These findings may complement the description of laboratory vulnerability but are insufficient to support treatment selection, risk stratification, or individualized management. Early readmission in HF is influenced jointly by cardiac disease severity, renal dysfunction, anemia, inflammation, nutritional status, and other comorbidities (33, 34), several of which also affect exercise capacity, functional recovery, and rehabilitation tolerance. Frailty, dietary and inflammatory factors, precision nutrition, outpatient follow-up, and transitional care may further influence functional recovery and postdischarge healthcare utilization in patients with HF (35–39).

Several limitations should be acknowledged. The single-center design and unequal proportions of complete cases across LVEF categories may have introduced selection bias; although the full-cohort imputation analyses yielded directionally consistent findings, this bias cannot be excluded. In addition, the NYHA class in this study was derived from the initial assessment upon admission and may be influenced by anemia, pulmonary disease, obesity, frailty, and other non cardiac factors, and may change with stable condition and diuretic treatment during hospitalization. Therefore, NYHA class III–IV were only used as clinical anchors for severe functional limitation in this study and should not be interpreted as specific indicators of heart failure severity, nor as independent biological validation for phenogroups. Readmission ascertainment captured only events at Beijing Anzhen Hospital, and readmissions to other hospitals may have been missed. In-hospital deaths were excluded, and only patients with a follow-up record at this hospital more than 30 days after discharge were included. Although these records confirmed survival at 30 days among included patients, out-of-hospital events may still have been missed and survivor-selection bias may remain, potentially leading to underestimation of readmission rates and conservative association estimates. The readmission findings should therefore be interpreted as associations with same-center events rather than as the complete 30-day all-cause readmission risk. Kidney function and hemoglobin were used to construct the phenogroups and were not repeatedly adjusted for in the outcome models. Previous HF hospitalization, HF etiology, atrial fibrillation, and discharge medications were not fully incorporated, and residual confounding may therefore remain. Future studies should integrate more complete information on lifestyle factors, pharmacologic therapy, and postdischarge management to further validate these phenogroups and their clinical implications.

## 5 Conclusion

Three phenogroups identified from routine admission laboratory measurements revealed substantial heterogeneity in inflammatory, nutritional, hematologic, and cardiorenal status among hospitalized patients with HF. After adjustment for continuous LVEF and NT-proBNP, the two vulnerable phenogroups remained associated with severe functional limitation and 30-day same-center all-cause unplanned readmission, with directionally consistent associations in the full-cohort and K = 2 sensitivity analyses. ALI and PNI reflect laboratory-derived inflammatory–nutritional vulnerability but cannot replace formal nutritional assessment or establish incremental predictive value. These findings are exploratory and require validation in prospective multicenter cohorts.

## Data Availability

The raw data supporting the conclusions of this article will be made available by the authors, without undue reservation.

## Glossary

ALI: Advanced Lung Cancer Inflammation Index
ARI: Adjusted Rand Index
AUC: Area Under the Receiver Operating Characteristic Curve
BIC: Bayesian Information Criterion
BMI: Body Mass Index
CALLY: C-Reactive Protein–Albumin–Lymphocyte Score
CH: Calinski–Harabasz
CI: Confidence Interval
CONUT: Controlling Nutritional Status
DBSCAN: Density-Based Spatial Clustering of Applications With Noise
eGFR: Estimated Glomerular Filtration Rate
GMM: Gaussian Mixture Model
HALP: Hemoglobin–Albumin–Lymphocyte–Platelet Index
HDL-C: High-Density Lipoprotein Cholesterol
HF: Heart Failure
hsCAR: High-Sensitivity C-Reactive Protein-to-Albumin Ratio
hs-CRP: High-Sensitivity C-Reactive Protein
IQR: Interquartile Range
LCR: Lymphocyte-to-C-Reactive Protein Ratio
LVEF: Left Ventricular Ejection Fraction
MAR: Monocyte-to-Albumin Ratio
NAR: Neutrophil-to-Albumin Ratio
NMI: Normalized Mutual Information
NPS: Naples Prognostic Score
NT-proBNP: N-Terminal Pro-B-Type Natriuretic Peptide
NYHA: New York Heart Association
OR: Odds Ratio
PC: Principal Component
PCA: Principal Component Analysis
PNI: Prognostic Nutritional Index
RAR: Red Cell Distribution Width-to-Albumin Ratio
RCS: Restricted Cubic Spline
RDW-CV: Red Blood Cell Distribution Width–Coefficient of Variation
SD: Standard Deviation
TC: Total Cholesterol
TG: Triglycerides
