# Supplementary material for: Laboratory-derived inflammatory–nutritional exploratory phenogroups and their associations with functional limitation and 30-day same-center all-cause unplanned readmission in patients with heart failure
Source: Front Nutr. 2026 Oct 9;13:1985953. doi: 10.3389/fnut.2026.1985953 (PMC13690677; doi:10.3389/fnut.2026.1985953)
Supplement: Supplementary file 1 [file Supplementary_file_1.DOCX]

**Supplementary materials (merged version)**

**Table S1. Missingness and Distribution of All 78 Source Variables**

| **Variable** | **Valid n** | **Missing n** | **Missing (%)** |
| --- | --- | --- | --- |
| LVEF | 1,588 | 0 | 0 |
| Hypertension | 1,588 | 0 | 0 |
| Diabetes mellitus | 1,588 | 0 | 0 |
| Dyslipidemia | 1,588 | 0 | 0 |
| Severe functional limitation | 1,588 | 0 | 0 |
| EF phenotype | 1,588 | 0 | 0 |
| Sex | 1,588 | 0 | 0 |
| Age | 1,588 | 0 | 0 |
| BMI | 1,587 | 1 | 0.1 |
| WBC | 1,583 | 5 | 0.3 |
| RBC | 1,583 | 5 | 0.3 |
| Hemoglobin | 1,583 | 5 | 0.3 |
| Hematocrit | 1,583 | 5 | 0.3 |
| MCV | 1,583 | 5 | 0.3 |
| MCH | 1,583 | 5 | 0.3 |
| MCHC | 1,583 | 5 | 0.3 |
| Platelet | 1,583 | 5 | 0.3 |
| MPV | 1,583 | 5 | 0.3 |
| Neutrophil (%) | 1,583 | 5 | 0.3 |
| Lymphocyte (%) | 1,583 | 5 | 0.3 |
| Monocyte (%) | 1,583 | 5 | 0.3 |
| Eosinophil (%) | 1,583 | 5 | 0.3 |
| Basophil (%) | 1,583 | 5 | 0.3 |
| Neutrophil | 1,583 | 5 | 0.3 |
| Lymphocyte | 1,583 | 5 | 0.3 |
| Monocyte | 1,583 | 5 | 0.3 |
| Eosinophil | 1,583 | 5 | 0.3 |
| Basophil | 1,583 | 5 | 0.3 |
| Plateletcrit | 1,583 | 5 | 0.3 |
| PDW | 1,583 | 5 | 0.3 |
| RDW-SD | 1,583 | 5 | 0.3 |
| RDW-CV | 1,583 | 5 | 0.3 |
| P-LCR | 1,583 | 5 | 0.3 |
| NLR | 1,583 | 5 | 0.3 |
| PLR | 1,583 | 5 | 0.3 |
| Creatinine (Jaffe) | 847 | 741 | 46.7 |
| Prealbumin | 1,460 | 128 | 8.1 |
| Lp(a) | 1,300 | 288 | 18.1 |
| Homocysteine | 1,460 | 128 | 8.1 |
| hs-CRP | 1,460 | 128 | 8.1 |
| ALT | 1,458 | 130 | 8.2 |
| AST | 1,458 | 130 | 8.2 |
| Total protein | 1,457 | 131 | 8.2 |
| Albumin | 1,459 | 129 | 8.1 |
| Total bilirubin | 1,458 | 130 | 8.2 |
| Direct bilirubin | 1,458 | 130 | 8.2 |
| ALP | 1,459 | 129 | 8.1 |
| GGT | 1,459 | 129 | 8.1 |
| Total bile acid | 1,460 | 128 | 8.1 |
| Cholinesterase | 1,459 | 129 | 8.1 |
| Urea | 1,460 | 128 | 8.1 |
| Uric acid | 1,459 | 129 | 8.1 |
| Glucose | 1,460 | 128 | 8.1 |
| CK | 1,458 | 130 | 8.2 |
| LDH | 1,458 | 130 | 8.2 |
| Triglycerides | 1,458 | 130 | 8.2 |
| Total cholesterol | 1,458 | 130 | 8.2 |
| HDL-C | 1,458 | 130 | 8.2 |
| LDL-C | 1,458 | 130 | 8.2 |
| Calcium | 1,460 | 128 | 8.1 |
| Phosphate | 1,460 | 128 | 8.1 |
| Magnesium | 1,460 | 128 | 8.1 |
| Sodium | 1,459 | 129 | 8.1 |
| Potassium | 1,459 | 129 | 8.1 |
| Chloride | 1,460 | 128 | 8.1 |
| Total CO2 | 1,460 | 128 | 8.1 |
| Albumin/globulin ratio | 1,457 | 131 | 8.2 |
| Anion gap | 1,459 | 129 | 8.1 |
| Glycated albumin (%) | 1,460 | 128 | 8.1 |
| Albumin (g/dL) | 1,460 | 128 | 8.1 |
| Glycated albumin (g/dL) | 1,460 | 128 | 8.1 |
| Free fatty acids | 1,308 | 280 | 17.6 |
| sdLDL-C | 1,308 | 280 | 17.6 |
| eGFR | 1,458 | 130 | 8.2 |
| non-HDL-C | 1,458 | 130 | 8.2 |
| CK-MB | 1,346 | 242 | 15.2 |
| RLP-C | 1,458 | 130 | 8.2 |
| NT-proBNP | 1,588 | 0 | 0 |

**Notes:** Missingness was assessed in the full study cohort (N=1,588). For each variable, valid n plus missing n equals 1,588, and missing percentages use 1,588 as the denominator.

**Abbreviations:** ALP, alkaline phosphatase; ALT, alanine aminotransferase; AST, aspartate aminotransferase; BMI, body mass index; CK, creatine kinase; CK-MB, creatine kinase-myocardial band; CO2, carbon dioxide; EF, ejection fraction; eGFR, estimated glomerular filtration rate; GGT, gamma-glutamyl transferase; HDL-C, high-density lipoprotein cholesterol; hs-CRP, high-sensitivity C-reactive protein; LDH, lactate dehydrogenase; LDL-C, low-density lipoprotein cholesterol; Lp(a), lipoprotein(a); LVEF, left ventricular ejection fraction; MCH, mean corpuscular hemoglobin; MCHC, mean corpuscular hemoglobin concentration; MCV, mean corpuscular volume; MPV, mean platelet volume; NLR, neutrophil-to-lymphocyte ratio; non-HDL-C, non-high-density lipoprotein cholesterol; NT-proBNP, N-terminal pro-B-type natriuretic peptide; P-LCR, platelet large cell ratio; PDW, platelet distribution width; PLR, platelet-to-lymphocyte ratio; RBC, red blood cell; RDW-CV, red blood cell distribution width-coefficient of variation; RDW-SD, red blood cell distribution width-standard deviation; RLP-C, remnant-like particle cholesterol; sdLDL-C, small dense low-density lipoprotein cholesterol; WBC, white blood cell.

**Table S2. Formulae and Units of Nutrition Indices**

| **Index** | **Formula** | **Reported unit** |
| --- | --- | --- |
| PNI | Albumin (g/L) + 5 × lymphocyte count (10^9/L) | index units |
| CONUT score | Albumin points (0/2/4/6) + total-cholesterol points (0/1/2/3) + lymphocyte points (0/1/2/3) | score, 0–12 |
| HALP | Hemoglobin (g/L) × albumin (g/L) × lymphocyte count (10^9/L) / platelet count (10^9/L) | unit-dependent index |
| hsCAR | hs-CRP (mg/L) / albumin (g/L) | mg/g |
| ALI | BMI (kg/m^2) × albumin (g/dL) / NLR | kg/m^2·g/dL |
| CALLY | Albumin (g/L) × lymphocyte count (10^9/L) / hs-CRP (mg/L) | unit-dependent index |
| NPS | I[albumin <40 g/L] + I[TC <=4.66 mmol/L] + I[NLR >2.96] + I[LMR <4.44] | score, 0–4 |
| RAR | RDW-CV (%) / albumin (g/L) | %·L/g |
| NAR | Neutrophil count (10^9/L) / albumin (g/L) | 10^9/g |
| MAR | Monocyte count (10^9/L) / albumin (g/L) | 10^9/g |
| LCR | Lymphocyte count (10^9/L) / hs-CRP (mg/L) | 10^9/mg |

**Notes:** Formulae were evaluated using the units shown.

**Abbreviations:** ALI, advanced lung cancer inflammation index; BMI, body mass index; CALLY, C-reactive protein-albumin-lymphocyte score; CONUT, Controlling Nutritional Status; HALP, hemoglobin-albumin-lymphocyte-platelet index; hsCAR, high-sensitivity C-reactive protein-to-albumin ratio; hs-CRP, high-sensitivity C-reactive protein; LCR, lymphocyte-to-C-reactive protein ratio; LMR, lymphocyte-to-monocyte ratio; MAR, monocyte-to-albumin ratio; NAR, neutrophil-to-albumin ratio; NLR, neutrophil-to-lymphocyte ratio; NPS, Naples prognostic score; PNI, prognostic nutritional index; RAR, red cell distribution width-to-albumin ratio; RDW-CV, red blood cell distribution width-coefficient of variation; TC, total cholesterol.

**Table S3. Complete Screening Results for K-Means, Gaussian Mixture Models, and DBSCAN**

| **Method** | **Configuration** | **K** | **Covariance** | **eps** | **Minimum samples** | **Silhouette** | **Calinski-Harabasz** | **Davies-Bouldin** | **BIC** | **Clusters found** | **Cluster sizes** | **Smallest cluster (%)** | **Noise (%)** |
| --- | --- | --- | --- | --- | --- | --- | --- | --- | --- | --- | --- | --- | --- |
| K-means | K=2; n_init=100 | 2 |  |  |  | 0.126 | 153.5 | 2.941 |  | 2 | 1:908; 2:547 | 37.6 | 0 |
| K-means | K=3; n_init=100 | 3 |  |  |  | 0.107 | 135.4 | 2.622 |  | 3 | 1:425; 2:264; 3:766 | 18.1 | 0 |
| K-means | K=4; n_init=100 | 4 |  |  |  | 0.094 | 123 | 2.469 |  | 4 | 1:229; 2:279; 3:309; 4:638 | 15.7 | 0 |
| K-means | K=5; n_init=100 | 5 |  |  |  | 0.098 | 113.1 | 2.352 |  | 5 | 1:273; 2:173; 3:609; 4:134; 5:266 | 9.2 | 0 |
| K-means | K=6; n_init=100 | 6 |  |  |  | 0.071 | 105.3 | 2.398 |  | 6 | 1:304; 2:232; 3:405; 4:150; 5:128; 6:236 | 8.8 | 0 |
| Gaussian mixture model | K=2; covariance=full; n_init=10 | 2 | full |  |  | 0.107 | 115.5 | 3.433 | 58530.7 | 2 | 1:599; 2:856 | 41.2 | 0 |
| Gaussian mixture model | K=3; covariance=full; n_init=10 | 3 | full |  |  | 0.056 | 97.4 | 3.282 | 58588.4 | 3 | 1:387; 2:430; 3:638 | 26.6 | 0 |
| Gaussian mixture model | K=4; covariance=full; n_init=10 | 4 | full |  |  | 0.053 | 85.4 | 3.104 | 59045.2 | 4 | 1:255; 2:578; 3:385; 4:237 | 16.3 | 0 |
| Gaussian mixture model | K=5; covariance=full; n_init=10 | 5 | full |  |  | 0.007 | 66.7 | 3.435 | 59583.8 | 5 | 1:362; 2:353; 3:286; 4:153; 5:301 | 10.5 | 0 |
| Gaussian mixture model | K=6; covariance=full; n_init=10 | 6 | full |  |  | 0.019 | 64.6 | 3.352 | 60216.6 | 6 | 1:402; 2:317; 3:145; 4:256; 5:91; 6:244 | 6.3 | 0 |
| Gaussian mixture model | K=2; covariance=tied; n_init=10 | 2 | tied |  |  | 0.16 | 127.8 | 2.745 | 59189.7 | 2 | 1:1161; 2:294 | 20.2 | 0 |
| Gaussian mixture model | K=3; covariance=tied; n_init=10 | 3 | tied |  |  | 0.121 | 98.6 | 2.525 | 58760.7 | 3 | 1:1095; 2:110; 3:250 | 7.6 | 0 |
| Gaussian mixture model | K=4; covariance=tied; n_init=10 | 4 | tied |  |  | 0.118 | 92.6 | 2.395 | 58472 | 4 | 1:123; 2:106; 3:1001; 4:225 | 7.3 | 0 |
| Gaussian mixture model | K=5; covariance=tied; n_init=10 | 5 | tied |  |  | 0.117 | 85.6 | 2.279 | 58387.8 | 5 | 1:125; 2:223; 3:931; 4:123; 5:53 | 3.6 | 0 |
| Gaussian mixture model | K=6; covariance=tied; n_init=10 | 6 | tied |  |  | 0.089 | 77.6 | 2.304 | 58473.8 | 6 | 1:864; 2:224; 3:120; 4:70; 5:124; 6:53 | 3.6 | 0 |
| Gaussian mixture model | K=2; covariance=diag; n_init=10 | 2 | diag |  |  | 0.126 | 123 | 3.339 | 60013 | 2 | 1:568; 2:887 | 39 | 0 |
| Gaussian mixture model | K=3; covariance=diag; n_init=10 | 3 | diag |  |  | 0.042 | 103 | 3.157 | 59162.8 | 3 | 1:562; 2:398; 3:495 | 27.4 | 0 |
| Gaussian mixture model | K=4; covariance=diag; n_init=10 | 4 | diag |  |  | 0.042 | 90.3 | 3.096 | 58830.6 | 4 | 1:472; 2:244; 3:268; 4:471 | 16.8 | 0 |
| Gaussian mixture model | K=5; covariance=diag; n_init=10 | 5 | diag |  |  | 0.059 | 91.2 | 2.715 | 58534.1 | 5 | 1:239; 2:321; 3:476; 4:195; 5:224 | 13.4 | 0 |
| Gaussian mixture model | K=6; covariance=diag; n_init=10 | 6 | diag |  |  | 0.027 | 82.9 | 2.798 | 58444.4 | 6 | 1:240; 2:314; 3:304; 4:164; 5:220; 6:213 | 11.3 | 0 |
| Gaussian mixture model | K=2; covariance=spherical; n_init=10 | 2 | spherical |  |  | 0.146 | 119.2 | 3.389 | 60307.3 | 2 | 1:946; 2:509 | 35 | 0 |
| Gaussian mixture model | K=3; covariance=spherical; n_init=10 | 3 | spherical |  |  | 0.106 | 111.7 | 2.885 | 59968.5 | 3 | 1:293; 2:296; 3:866 | 20.1 | 0 |
| Gaussian mixture model | K=4; covariance=spherical; n_init=10 | 4 | spherical |  |  | 0.097 | 106.3 | 2.52 | 59728.9 | 4 | 1:244; 2:781; 3:201; 4:229 | 13.8 | 0 |
| Gaussian mixture model | K=5; covariance=spherical; n_init=10 | 5 | spherical |  |  | 0.092 | 98.7 | 2.541 | 59518.7 | 5 | 1:179; 2:706; 3:189; 4:241; 5:140 | 9.6 | 0 |
| Gaussian mixture model | K=6; covariance=spherical; n_init=10 | 6 | spherical |  |  | 0.051 | 90.6 | 2.68 | 59344.8 | 6 | 1:443; 2:248; 3:231; 4:215; 5:127; 6:191 | 8.7 | 0 |
| DBSCAN | min_samples=10; k-distance q=0.700 |  |  | 3.145 | 10 |  |  |  |  | 1 | 1:1263 | 86.8 | 13.2 |
| DBSCAN | min_samples=10; k-distance q=0.750 |  |  | 3.258 | 10 |  |  |  |  | 1 | 1:1306 | 89.8 | 10.2 |
| DBSCAN | min_samples=10; k-distance q=0.800 |  |  | 3.435 | 10 |  |  |  |  | 1 | 1:1347 | 92.6 | 7.4 |
| DBSCAN | min_samples=10; k-distance q=0.850 |  |  | 3.58 | 10 |  |  |  |  | 1 | 1:1374 | 94.4 | 5.6 |
| DBSCAN | min_samples=10; k-distance q=0.900 |  |  | 3.826 | 10 |  |  |  |  | 1 | 1:1408 | 96.8 | 3.2 |
| DBSCAN | min_samples=10; k-distance q=0.925 |  |  | 3.987 | 10 |  |  |  |  | 1 | 1:1420 | 97.6 | 2.4 |
| DBSCAN | min_samples=10; k-distance q=0.950 |  |  | 4.243 | 10 |  |  |  |  | 1 | 1:1435 | 98.6 | 1.4 |
| DBSCAN | min_samples=10; k-distance q=0.975 |  |  | 4.645 | 10 |  |  |  |  | 1 | 1:1446 | 99.4 | 0.6 |
| DBSCAN | min_samples=15; k-distance q=0.700 |  |  | 3.281 | 15 |  |  |  |  | 1 | 1:1296 | 89.1 | 10.9 |
| DBSCAN | min_samples=15; k-distance q=0.750 |  |  | 3.395 | 15 |  |  |  |  | 1 | 1:1333 | 91.6 | 8.4 |
| DBSCAN | min_samples=15; k-distance q=0.800 |  |  | 3.555 | 15 |  |  |  |  | 1 | 1:1363 | 93.7 | 6.3 |
| DBSCAN | min_samples=15; k-distance q=0.850 |  |  | 3.733 | 15 |  |  |  |  | 1 | 1:1389 | 95.5 | 4.5 |
| DBSCAN | min_samples=15; k-distance q=0.900 |  |  | 4.018 | 15 |  |  |  |  | 1 | 1:1420 | 97.6 | 2.4 |
| DBSCAN | min_samples=15; k-distance q=0.925 |  |  | 4.167 | 15 |  |  |  |  | 1 | 1:1431 | 98.4 | 1.6 |
| DBSCAN | min_samples=15; k-distance q=0.950 |  |  | 4.389 | 15 |  |  |  |  | 1 | 1:1439 | 98.9 | 1.1 |
| DBSCAN | min_samples=15; k-distance q=0.975 |  |  | 4.858 | 15 |  |  |  |  | 1 | 1:1446 | 99.4 | 0.6 |
| DBSCAN | min_samples=20; k-distance q=0.700 |  |  | 3.374 | 20 |  |  |  |  | 1 | 1:1311 | 90.1 | 9.9 |
| DBSCAN | min_samples=20; k-distance q=0.750 |  |  | 3.5 | 20 |  |  |  |  | 1 | 1:1343 | 92.3 | 7.7 |
| DBSCAN | min_samples=20; k-distance q=0.800 |  |  | 3.661 | 20 |  |  |  |  | 1 | 1:1375 | 94.5 | 5.5 |
| DBSCAN | min_samples=20; k-distance q=0.850 |  |  | 3.855 | 20 |  |  |  |  | 1 | 1:1396 | 95.9 | 4.1 |
| DBSCAN | min_samples=20; k-distance q=0.900 |  |  | 4.127 | 20 |  |  |  |  | 1 | 1:1424 | 97.9 | 2.1 |
| DBSCAN | min_samples=20; k-distance q=0.925 |  |  | 4.292 | 20 |  |  |  |  | 1 | 1:1434 | 98.6 | 1.4 |
| DBSCAN | min_samples=20; k-distance q=0.950 |  |  | 4.514 | 20 |  |  |  |  | 1 | 1:1444 | 99.2 | 0.8 |
| DBSCAN | min_samples=20; k-distance q=0.975 |  |  | 4.993 | 20 |  |  |  |  | 1 | 1:1446 | 99.4 | 0.6 |
| DBSCAN | min_samples=30; k-distance q=0.700 |  |  | 3.528 | 30 |  |  |  |  | 1 | 1:1335 | 91.8 | 8.2 |
| DBSCAN | min_samples=30; k-distance q=0.750 |  |  | 3.653 | 30 |  |  |  |  | 1 | 1:1364 | 93.7 | 6.3 |
| DBSCAN | min_samples=30; k-distance q=0.800 |  |  | 3.824 | 30 |  |  |  |  | 1 | 1:1390 | 95.5 | 4.5 |
| DBSCAN | min_samples=30; k-distance q=0.850 |  |  | 4.023 | 30 |  |  |  |  | 1 | 1:1413 | 97.1 | 2.9 |
| DBSCAN | min_samples=30; k-distance q=0.900 |  |  | 4.289 | 30 |  |  |  |  | 1 | 1:1430 | 98.3 | 1.7 |
| DBSCAN | min_samples=30; k-distance q=0.925 |  |  | 4.465 | 30 |  |  |  |  | 1 | 1:1437 | 98.8 | 1.2 |
| DBSCAN | min_samples=30; k-distance q=0.950 |  |  | 4.704 | 30 |  |  |  |  | 1 | 1:1445 | 99.3 | 0.7 |
| DBSCAN | min_samples=30; k-distance q=0.975 |  |  | 5.173 | 30 |  |  |  |  | 1 | 1:1447 | 99.5 | 0.5 |
| DBSCAN | min_samples=45; k-distance q=0.700 |  |  | 3.681 | 45 |  |  |  |  | 1 | 1:1352 | 92.9 | 7.1 |
| DBSCAN | min_samples=45; k-distance q=0.750 |  |  | 3.814 | 45 |  |  |  |  | 1 | 1:1381 | 94.9 | 5.1 |
| DBSCAN | min_samples=45; k-distance q=0.800 |  |  | 3.969 | 45 |  |  |  |  | 1 | 1:1402 | 96.4 | 3.6 |
| DBSCAN | min_samples=45; k-distance q=0.850 |  |  | 4.191 | 45 |  |  |  |  | 1 | 1:1420 | 97.6 | 2.4 |
| DBSCAN | min_samples=45; k-distance q=0.900 |  |  | 4.466 | 45 |  |  |  |  | 1 | 1:1436 | 98.7 | 1.3 |
| DBSCAN | min_samples=45; k-distance q=0.925 |  |  | 4.626 | 45 |  |  |  |  | 1 | 1:1440 | 99 | 1 |
| DBSCAN | min_samples=45; k-distance q=0.950 |  |  | 4.91 | 45 |  |  |  |  | 1 | 1:1445 | 99.3 | 0.7 |
| DBSCAN | min_samples=45; k-distance q=0.975 |  |  | 5.388 | 45 |  |  |  |  | 1 | 1:1449 | 99.6 | 0.4 |

**Notes:** All methods used the same 1,455 adult complete cases and the same transformed, standardized 15-variable matrix. K-means used seed 20260830 and n_init=100; GMM used n_init=10.

DBSCAN was screened over 40 parameter combinations. None formed more than one cluster, so DBSCAN did not yield a valid phenotype solution for these data.

The lowest GMM BIC occurred for tied-covariance K=5, but its smallest group contained 53 patients (3.64%), below the prespecified 5% threshold.

K denotes the prespecified number of clusters; eps is the DBSCAN neighborhood radius; min_samples is the minimum number of observations required to form a dense region; n_init is the number of initializations; and q is the k-distance quantile used to derive eps.

**Abbreviations:** BIC, Bayesian information criterion; DBSCAN, density-based spatial clustering of applications with noise; GMM, Gaussian mixture model.

**Table S4. Five-Hundred-Replicate Bootstrap Stability of Candidate Cluster Solutions**

| **Method** | **K** | **Scope** | **Reference cluster** | **Successful bootstraps** | **Mean Jaccard** | **Median Jaccard** | **95% CI lower** | **95% CI upper** | **5th percentile** | **Minimum** | **Weakest cluster mean Jaccard** |
| --- | --- | --- | --- | --- | --- | --- | --- | --- | --- | --- | --- |
| K-means | 2 | cluster | 1 | 500 | 0.891 | 0.888 | 0.798 | 0.974 | 0.816 | 0.738 |  |
| K-means | 2 | cluster | 2 | 500 | 0.812 | 0.804 | 0.64 | 0.958 | 0.685 | 0.553 |  |
| K-means | 2 | overall mean across matched clusters |  | 500 | 0.852 | 0.846 | 0.72 | 0.966 | 0.752 | 0.654 | 0.812 |
| K-means | 3 | cluster | 1 | 500 | 0.8 | 0.82 | 0.58 | 0.93 | 0.615 | 0.276 |  |
| K-means | 3 | cluster | 2 | 500 | 0.784 | 0.806 | 0.528 | 0.921 | 0.595 | 0.216 |  |
| K-means | 3 | cluster | 3 | 500 | 0.883 | 0.893 | 0.772 | 0.957 | 0.788 | 0.661 |  |
| K-means | 3 | overall mean across matched clusters |  | 500 | 0.823 | 0.84 | 0.663 | 0.926 | 0.695 | 0.43 | 0.784 |
| K-means | 4 | cluster | 1 | 500 | 0.79 | 0.825 | 0.543 | 0.951 | 0.561 | 0.502 |  |
| K-means | 4 | cluster | 2 | 500 | 0.861 | 0.876 | 0.655 | 0.94 | 0.746 | 0.386 |  |
| K-means | 4 | cluster | 3 | 500 | 0.732 | 0.774 | 0.285 | 0.888 | 0.408 | 0.083 |  |
| K-means | 4 | cluster | 4 | 500 | 0.868 | 0.885 | 0.703 | 0.943 | 0.731 | 0.469 |  |
| K-means | 4 | overall mean across matched clusters |  | 500 | 0.813 | 0.835 | 0.547 | 0.92 | 0.635 | 0.425 | 0.732 |
| K-means | 5 | cluster | 1 | 500 | 0.728 | 0.741 | 0.426 | 0.895 | 0.538 | 0.155 |  |
| K-means | 5 | cluster | 2 | 500 | 0.568 | 0.651 | 0.021 | 0.926 | 0.043 | 0.012 |  |
| K-means | 5 | cluster | 3 | 500 | 0.746 | 0.827 | 0.396 | 0.947 | 0.416 | 0.361 |  |
| K-means | 5 | cluster | 4 | 500 | 0.839 | 0.899 | 0.45 | 0.97 | 0.504 | 0 |  |
| K-means | 5 | cluster | 5 | 500 | 0.839 | 0.85 | 0.68 | 0.934 | 0.716 | 0.571 |  |
| K-means | 5 | overall mean across matched clusters |  | 500 | 0.744 | 0.791 | 0.479 | 0.919 | 0.51 | 0.417 | 0.568 |
| GMM tied | 3 | cluster | 1 | 500 | 0.866 | 0.862 | 0.582 | 0.98 | 0.672 | 0.422 |  |
| GMM tied | 3 | cluster | 2 | 500 | 0.502 | 0.438 | 0 | 0.918 | 0.016 | 0 |  |
| GMM tied | 3 | cluster | 3 | 500 | 0.742 | 0.855 | 0.011 | 0.942 | 0.03 | 0 |  |
| GMM tied | 3 | overall mean across matched clusters |  | 500 | 0.703 | 0.667 | 0.338 | 0.939 | 0.398 | 0.287 | 0.502 |
| GMM tied | 4 | cluster | 1 | 500 | 0.699 | 0.848 | 0.021 | 0.944 | 0.032 | 0 |  |
| GMM tied | 4 | cluster | 2 | 500 | 0.311 | 0.082 | 0.009 | 0.856 | 0.017 | 0 |  |
| GMM tied | 4 | cluster | 3 | 500 | 0.829 | 0.849 | 0.498 | 0.959 | 0.576 | 0.367 |  |
| GMM tied | 4 | cluster | 4 | 500 | 0.802 | 0.847 | 0.063 | 0.92 | 0.233 | 0 |  |
| GMM tied | 4 | overall mean across matched clusters |  | 500 | 0.66 | 0.641 | 0.453 | 0.897 | 0.501 | 0.254 | 0.311 |
| GMM tied | 5 | cluster | 1 | 500 | 0.476 | 0.586 | 0.008 | 0.908 | 0.015 | 0 |  |
| GMM tied | 5 | cluster | 2 | 500 | 0.827 | 0.849 | 0.576 | 0.92 | 0.713 | 0.013 |  |
| GMM tied | 5 | cluster | 3 | 500 | 0.781 | 0.797 | 0.474 | 0.955 | 0.53 | 0.365 |  |
| GMM tied | 5 | cluster | 4 | 500 | 0.765 | 0.867 | 0.026 | 0.96 | 0.037 | 0 |  |
| GMM tied | 5 | cluster | 5 | 500 | 0.411 | 0.491 | 0.008 | 0.799 | 0.013 | 0 |  |
| GMM tied | 5 | overall mean across matched clusters |  | 500 | 0.652 | 0.637 | 0.446 | 0.877 | 0.468 | 0.241 | 0.411 |

**Notes:** Cluster labels in each bootstrap sample were matched to the reference solution before Jaccard calculation. All candidate analyses completed 500 replicates with no failures.

K-means K=3 achieved an overall mean Jaccard of 0.823 and a weakest cluster mean of 0.784; tied-covariance GMM K=3 achieved 0.703 and 0.502, respectively.

**Abbreviations:** CI, confidence interval; GMM, Gaussian mixture model.

**Table S5. Full-cohort K=3 missing-data sensitivity analyses**

**Panel A. Full-cohort K=3 models after 30 multiple imputations**

| **Outcome** | **Comparison** | **N** | **Events** | **OR (95% CI)** | **P value** |
| --- | --- | --- | --- | --- | --- |
| NYHA class III–IV | Cluster 1 vs Cluster 3 | 1,588 | 379 | 3.474 (2.534–4.764) | <0.001 |
| NYHA class III–IV | Cluster 2 vs Cluster 3 | 1,588 | 379 | 4.790 (3.378–6.794) | <0.001 |
| 30-day same-center all-cause unplanned readmission | Cluster 1 vs Cluster 3 | 1,588 | 214 | 1.810 (1.254–2.614) | 0.002 |
| 30-day same-center all-cause unplanned readmission | Cluster 2 vs Cluster 3 | 1,588 | 214 | 2.212 (1.489–3.285) | <0.001 |

**Panel B. Median-imputation K=3 sensitivity analysis**

| **Outcome** | **Comparison** | **N** | **Events** | **OR (95% CI)** | **P value** |
| --- | --- | --- | --- | --- | --- |
| NYHA class III–IV | Cluster 1 vs Cluster 3 | 1,588 | 379 | 4.168 (3.089–5.624) | <0.001 |
| NYHA class III–IV | Cluster 2 vs Cluster 3 | 1,588 | 379 | 4.400 (3.166–6.114) | <0.001 |
| 30-day same-center all-cause unplanned readmission | Cluster 1 vs Cluster 3 | 1,588 | 214 | 1.825 (1.271–2.621) | 0.001 |
| 30-day same-center all-cause unplanned readmission | Cluster 2 vs Cluster 3 | 1,588 | 214 | 2.314 (1.591–3.366) | <0.001 |

**Panel C. Clustering stability and cluster sizes**

| **Sensitivity analysis** | **Metric** | **Value** |
| --- | --- | --- |
| Median imputation | ARI on complete-case overlap | 0.936 |
| Median imputation | NMI on complete-case overlap | 0.888 |
| Median imputation | Aligned agreement, % | 97.7 |
| Median imputation | Cluster sizes | 410 / 322 / 856 |
| Multiple imputation | Mean ARI on complete-case overlap | 0.931 |
| Multiple imputation | Minimum ARI on complete-case overlap | 0.843 |
| Multiple imputation | Mean NMI on complete-case overlap | 0.881 |
| Multiple imputation | Mean aligned agreement, % | 97.5 |
| Multiple imputation | Mean patient modal assignment proportion | 0.954 |
| Multiple imputation | Patients with modal assignment proportion <0.80, n | 161 |
| Multiple imputation | Mean cluster sizes | 457.2 / 307.6 / 823.2 |

**Notes:** Cluster 3 is the reference group. ORs were estimated using logistic regression with HC3 robust standard errors. The NYHA model adjusted for age, sex, hypertension, diabetes, dyslipidemia, continuous LVEF, and log-transformed NT-proBNP; the readmission model additionally adjusted for NYHA class III–IV.

Multiple imputation used 30 outcome-blind imputations; neither NYHA class nor 30-day readmission was included in the imputation procedure. K=3 clustering was refitted and label-aligned within each imputed dataset, and coefficients were pooled using Rubin's rules. Median imputation was evaluated separately.

K denotes the number of clusters; N denotes the analysis sample size; events denotes the number of participants with the specified outcome.

**Abbreviations:** ARI, adjusted Rand index; CI, confidence interval; HC3, heteroskedasticity-consistent type 3; LVEF, left ventricular ejection fraction; NMI, normalized mutual information; NT-proBNP, N-terminal pro-B-type natriuretic peptide; NYHA, New York Heart Association; OR, odds ratio.

**Table S6. K=2 phenogroup sensitivity analysis**

**Panel A. Characteristics of the K=2 phenogroups**

| **Variable** | **Summary** | **Cluster 1 (n=547)** | **Cluster 2 (n=908)** | **P value** |
| --- | --- | --- | --- | --- |
| BMI | Mean ± SD | 23.45 ± 3.13 | 23.61 ± 3.13 | 0.344 |
| Albumin | Mean ± SD | 40.90 ± 4.66 | 44.36 ± 3.43 | <0.001 |
| Hemoglobin | Mean ± SD | 121.31 ± 27.34 | 145.76 ± 20.90 | <0.001 |
| RDW-CV | Median [IQR] | 14.80 [13.40, 16.20] | 13.30 [12.80, 14.00] | <0.001 |
| Lymphocyte | Median [IQR] | 1.11 [0.74, 1.55] | 1.69 [1.29, 2.14] | <0.001 |
| Neutrophil | Median [IQR] | 5.27 [4.01, 7.93] | 4.14 [3.29, 5.31] | <0.001 |
| Monocyte | Median [IQR] | 0.48 [0.37, 0.64] | 0.41 [0.33, 0.53] | <0.001 |
| hs-CRP | Median [IQR] | 3.76 [1.38, 12.35] | 1.01 [0.56, 2.30] | <0.001 |
| Platelet | Median [IQR] | 173.00 [127.50, 228.50] | 195.00 [160.00, 236.25] | <0.001 |
| TC | Median [IQR] | 3.66 [3.00, 4.58] | 3.71 [3.11, 4.55] | 0.277 |
| TG | Median [IQR] | 1.40 [1.01, 1.95] | 1.27 [0.98, 1.79] | 0.010 |
| HDL-C | Mean ± SD | 1.00 ± 0.26 | 1.18 ± 0.29 | <0.001 |
| Glucose | Median [IQR] | 5.76 [4.92, 7.38] | 5.55 [4.94, 6.51] | 0.007 |
| eGFR | Mean ± SD | 66.81 ± 25.69 | 83.80 ± 20.51 | <0.001 |
| Uric acid | Median [IQR] | 399.70 [323.20, 494.35] | 308.75 [251.90, 371.23] | <0.001 |

**Panel B. Enhanced-adjustment outcome models**

| **Outcome** | **Comparison** | **N** | **Events** | **OR (95% CI)** | **P value** |
| --- | --- | --- | --- | --- | --- |
| NYHA class III–IV | Cluster 1 vs Cluster 2 | 1,455 | 371 | 5.279 (4.025–6.924) | <0.001 |
| 30-day same-center all-cause unplanned readmission | Cluster 1 vs Cluster 2 | 1,455 | 196 | 2.231 (1.596–3.119) | <0.001 |

**Panel C. Comparison of clustering metrics**

| **Solution** | **N** | **Cluster sizes** | **Silhouette** | **Calinski–Harabasz** | **Davies–Bouldin** |
| --- | --- | --- | --- | --- | --- |
| K-means K=2 | 1,455 | 547 / 908 | 0.126 | 153.46 | 2.941 |
| K-means K=3 | 1,455 | 425 / 264 / 766 | 0.107 | 135.38 | 2.622 |

**Notes:** K=2 clustering used the same 1,455 complete cases, 15 clustering variables, transformations, and standardization as the primary analysis, with n_init=100, max_iter=1,000, and random seed 20260830. Cluster 2 is the reference group.

ORs were estimated using logistic regression with HC3 robust standard errors. The NYHA model adjusted for age, sex, hypertension, diabetes, dyslipidemia, continuous LVEF, and log-transformed NT-proBNP; the readmission model additionally adjusted for NYHA class III–IV.

K denotes the number of clusters; N denotes the analysis sample size; n denotes the number of participants in the specified cluster.

**Abbreviations:** BMI, body mass index; CI, confidence interval; eGFR, estimated glomerular filtration rate; HC3, heteroskedasticity-consistent type 3; HDL-C, high-density lipoprotein cholesterol; hs-CRP, high-sensitivity C-reactive protein; IQR, interquartile range; LVEF, left ventricular ejection fraction; NT-proBNP, N-terminal pro-B-type natriuretic peptide; NYHA, New York Heart Association; OR, odds ratio; RDW-CV, red blood cell distribution width-coefficient of variation; SD, standard deviation; TC, total cholesterol; TG, triglycerides.

**Table S7. Complete-case selection and missing-data patterns**

**Panel A. Complete-case inclusion by LVEF stratum**

| **Population** | **N** | **Complete cases, n** | **Complete, %** | **Incomplete cases, n** | **Incomplete, %** |
| --- | --- | --- | --- | --- | --- |
| LVEF <50% | 1,332 | 1,327 | 99.6 | 5 | 0.4 |
| LVEF ≥50% | 256 | 128 | 50.0 | 128 | 50.0 |
| Overall | 1,588 | 1,455 | 91.6 | 133 | 8.4 |

**Panel B. Overall comparison of complete and incomplete cases**

| **Characteristic** | **Summary** | **Complete cases** | **Incomplete cases** | **Valid n, complete** | **Valid n, incomplete** | **P value** |
| --- | --- | --- | --- | --- | --- | --- |
| Age, years | Mean ± SD | 60.00 ± 14.20 | 60.35 ± 12.38 | 1,455 | 133 | 0.758 |
| Male sex | n (%) | 1,076 (74.0%) | 104 (78.2%) | 1,455 | 133 | 0.284 |
| BMI, kg/m² | Mean ± SD | 23.55 ± 3.13 | 24.07 ± 3.31 | 1,455 | 132 | 0.085 |
| LVEF, % | Mean ± SD | 33.54 ± 11.94 | 59.68 ± 8.00 | 1,455 | 133 | <0.001 |
| Hemoglobin, g/L | Mean ± SD | 136.56 ± 26.33 | 127.30 ± 23.28 | 1,455 | 128 | <0.001 |
| NT-proBNP, pg/mL | Median [IQR] | 1192.15 [443.77, 3236.64] | 859.00 [309.19, 1883.09] | 1,455 | 133 | 0.031 |
| Hypertension | n (%) | 807 (55.5%) | 79 (59.4%) | 1,455 | 133 | 0.382 |
| Diabetes mellitus | n (%) | 558 (38.4%) | 54 (40.6%) | 1,455 | 133 | 0.610 |
| Dyslipidemia | n (%) | 631 (43.4%) | 62 (46.6%) | 1,455 | 133 | 0.470 |
| NYHA class III–IV | n (%) | 371 (25.5%) | 8 (6.0%) | 1,455 | 133 | <0.001 |
| 30-day same-center all-cause unplanned readmission | n (%) | 196 (13.5%) | 18 (13.5%) | 1,455 | 133 | 0.984 |

**Panel C. Comparison restricted to patients with LVEF ≥50%**

| **Characteristic** | **Summary** | **Complete cases** | **Incomplete cases** | **Valid n, complete** | **Valid n, incomplete** | **P value** |
| --- | --- | --- | --- | --- | --- | --- |
| Age, years | Mean ± SD | 60.41 ± 12.09 | 60.08 ± 12.46 | 128 | 128 | 0.831 |
| Male sex | n (%) | 98 (76.6%) | 99 (77.3%) | 128 | 128 | 0.882 |
| BMI, kg/m² | Mean ± SD | 23.89 ± 2.95 | 24.11 ± 3.34 | 128 | 128 | 0.580 |
| LVEF, % | Mean ± SD | 60.01 ± 6.35 | 60.63 ± 6.37 | 128 | 128 | 0.432 |
| Hemoglobin, g/L | Mean ± SD | 132.09 ± 26.07 | 126.79 ± 23.38 | 128 | 123 | 0.091 |
| NT-proBNP, pg/mL | Median [IQR] | 1700.00 [546.25, 3875.50] | 863.00 [313.55, 1970.57] | 128 | 128 | 0.025 |
| Hypertension | n (%) | 78 (60.9%) | 76 (59.4%) | 128 | 128 | 0.798 |
| Diabetes mellitus | n (%) | 52 (40.6%) | 53 (41.4%) | 128 | 128 | 0.899 |
| Dyslipidemia | n (%) | 61 (47.7%) | 59 (46.1%) | 128 | 128 | 0.802 |
| NYHA class III–IV | n (%) | 79 (61.7%) | 8 (6.2%) | 128 | 128 | <0.001 |
| 30-day same-center all-cause unplanned readmission | n (%) | 15 (11.7%) | 18 (14.1%) | 128 | 128 | 0.576 |

**Panel D. Missingness in the 15 clustering variables and common joint patterns**

| **Variable** | **Population** | **N** | **Missing, n** | **Missing, %** |
| --- | --- | --- | --- | --- |
| BMI | Overall | 1,588 | 1 | 0.1 |
| BMI | LVEF <50% | 1,332 | 1 | 0.1 |
| BMI | LVEF ≥50% | 256 | 0 | 0.0 |
| Albumin | Overall | 1,588 | 129 | 8.1 |
| Albumin | LVEF <50% | 1,332 | 2 | 0.2 |
| Albumin | LVEF ≥50% | 256 | 127 | 49.6 |
| Hemoglobin | Overall | 1,588 | 5 | 0.3 |
| Hemoglobin | LVEF <50% | 1,332 | 0 | 0.0 |
| Hemoglobin | LVEF ≥50% | 256 | 5 | 2.0 |
| RDW-CV | Overall | 1,588 | 5 | 0.3 |
| RDW-CV | LVEF <50% | 1,332 | 0 | 0.0 |
| RDW-CV | LVEF ≥50% | 256 | 5 | 2.0 |
| Lymphocyte | Overall | 1,588 | 5 | 0.3 |
| Lymphocyte | LVEF <50% | 1,332 | 0 | 0.0 |
| Lymphocyte | LVEF ≥50% | 256 | 5 | 2.0 |
| Neutrophil | Overall | 1,588 | 5 | 0.3 |
| Neutrophil | LVEF <50% | 1,332 | 0 | 0.0 |
| Neutrophil | LVEF ≥50% | 256 | 5 | 2.0 |
| Monocyte | Overall | 1,588 | 5 | 0.3 |
| Monocyte | LVEF <50% | 1,332 | 0 | 0.0 |
| Monocyte | LVEF ≥50% | 256 | 5 | 2.0 |
| hs-CRP | Overall | 1,588 | 128 | 8.1 |
| hs-CRP | LVEF <50% | 1,332 | 1 | 0.1 |
| hs-CRP | LVEF ≥50% | 256 | 127 | 49.6 |
| Platelet | Overall | 1,588 | 5 | 0.3 |
| Platelet | LVEF <50% | 1,332 | 0 | 0.0 |
| Platelet | LVEF ≥50% | 256 | 5 | 2.0 |
| TC | Overall | 1,588 | 130 | 8.2 |
| TC | LVEF <50% | 1,332 | 2 | 0.2 |
| TC | LVEF ≥50% | 256 | 128 | 50.0 |
| TG | Overall | 1,588 | 130 | 8.2 |
| TG | LVEF <50% | 1,332 | 2 | 0.2 |
| TG | LVEF ≥50% | 256 | 128 | 50.0 |
| HDL-C | Overall | 1,588 | 130 | 8.2 |
| HDL-C | LVEF <50% | 1,332 | 2 | 0.2 |
| HDL-C | LVEF ≥50% | 256 | 128 | 50.0 |
| Glucose | Overall | 1,588 | 128 | 8.1 |
| Glucose | LVEF <50% | 1,332 | 1 | 0.1 |
| Glucose | LVEF ≥50% | 256 | 127 | 49.6 |
| eGFR | Overall | 1,588 | 130 | 8.2 |
| eGFR | LVEF <50% | 1,332 | 2 | 0.2 |
| eGFR | LVEF ≥50% | 256 | 128 | 50.0 |
| Uric acid | Overall | 1,588 | 129 | 8.1 |
| Uric acid | LVEF <50% | 1,332 | 1 | 0.1 |
| Uric acid | LVEF ≥50% | 256 | 128 | 50.0 |

| **Common joint missing-variable patterns** |  |  |  |  |
| --- | --- | --- | --- | --- |
| Missing-variable pattern | N | % | LVEF <50%, n | LVEF ≥50%, n |
| Complete | 1,455 | 91.6 | 1,327 | 128 |
| Albumin; hs-CRP; TC; TG; HDL-C; Glucose; eGFR; Uric acid | 123 | 7.7 | 1 | 122 |
| Albumin; Hemoglobin; RDW-CV; Lymphocyte; Neutrophil; Monocyte; hs-CRP; Platelet; TC; TG; HDL-C; Glucose; eGFR; Uric acid | 5 | 0.3 | 0 | 5 |
| TC; TG; HDL-C | 1 | 0.1 | 1 | 0 |
| BMI | 1 | 0.1 | 1 | 0 |
| eGFR | 1 | 0.1 | 1 | 0 |
| Albumin | 1 | 0.1 | 1 | 0 |
| TC; TG; HDL-C; eGFR; Uric acid | 1 | 0.1 | 0 | 1 |

**Notes:** Complete cases had nonmissing values for all 15 clustering variables. The analytic cohort comprised 1,455 complete cases and 133 incomplete cases (1,588 total).

N denotes the population size and n denotes the subgroup or nonmissing count, as indicated.

Continuous variables were compared using Welch's t test or the Mann-Whitney U test according to distribution; categorical variables were compared using the chi-square or Fisher exact test, as appropriate. P values were not adjusted for multiple comparisons.

**Abbreviations:** BMI, body mass index; eGFR, estimated glomerular filtration rate; HDL-C, high-density lipoprotein cholesterol; hs-CRP, high-sensitivity C-reactive protein; IQR, interquartile range; LVEF, left ventricular ejection fraction; NT-proBNP, N-terminal pro-B-type natriuretic peptide; NYHA, New York Heart Association; RDW-CV, red blood cell distribution width-coefficient of variation; SD, standard deviation; TC, total cholesterol; TG, triglycerides.

Figure S1. Spearman correlation structure between candidate variables

**Abbreviations:** BMI, body mass index; eGFR, estimated glomerular filtration rate; HDL-C, high-density lipoprotein cholesterol; hs-CRP, high-sensitivity C-reactive protein; RDW-CV, red blood cell distribution width-coefficient of variation; TC, total cholesterol; TG, triglycerides.

Figure S2. Subgroup analysis
